# Peculiar protrusions along tanycyte processes face diverse neural and nonneural cell types in the hypothalamic parenchyma

**DOI:** 10.1002/cne.24965

**Published:** 2020-06-24

**Authors:** Roxane Pasquettaz, Irina Kolotuev, Antoine Rohrbach, Cathy Gouelle, Luc Pellerin, Fanny Langlet

**Affiliations:** ^1^ Center for Integrative Genomics, Faculty of Biology and Medicine University of Lausanne Lausanne Switzerland; ^2^ Electron Microscopy Facility, Faculty of Biology and Medicine University of Lausanne Lausanne Switzerland; ^3^ Department of Physiology, Faculty of Biology and Medicine University of Lausanne Lausanne Switzerland; ^4^ Centre de Résonance Magnétique des Systèmes Biologiques UMR5536 CNRS, LabEx TRAIL‐IBIO, Université de Bordeaux Bordeaux Cedex France; ^5^ Inserm U1082 Universite de Poitiers Poitiers Cedex France; ^6^ Present address: Inserm U1082 Université de Poitiers Poitiers Cedex 86021 France

**Keywords:** arcuate neurons, electron microscopy, fluorescence microscopy, glia–neuron communication, hypothalamus, tanycytes

## Abstract

Tanycytes are highly specialized ependymal cells that line the bottom and the lateral walls of the third ventricle. In contact with the cerebrospinal fluid through their cell bodies, they send processes into the arcuate nucleus, the ventromedial nucleus, and the dorsomedial nucleus of the hypothalamus. In the present work, we combined transgenic and immunohistochemical approaches to investigate the neuroanatomical associations between tanycytes and neural cells present in the hypothalamic parenchyma, in particular in the arcuate nucleus. The specific expression of tdTomato in tanycytes first allowed the observation of peculiar subcellular protrusions along tanycyte processes and at their endfeet such as spines, swelling, en passant boutons, boutons, or claws. Interestingly, these protrusions contact different neural cells in the brain parenchyma including blood vessels and neurons, and in particular NPY and POMC neurons in the arcuate nucleus. Using both fluorescent and electron microscopy, we finally observed that these tanycyte protrusions contain ribosomes, mitochondria, diverse vesicles, and transporters, suggesting dense tanycyte/neuron and tanycyte/blood vessel communications. Altogether, our results lay the neuroanatomical basis for tanycyte/neural cell interactions, which will be useful to further understand cell‐to‐cell communications involved in the regulation of neuroendocrine functions.

## Graphical abstract

Using TAT‐Cre‐mediated transduction of tdTomato in tanycytes, this study morphologically describes peculiar protrusions along tanycyte processes. Combining tdTomato filling, fluorescence immunohistochemistry and electron microscopy, the authors show in 3D reconstructions the diverse tanycyte partners as well as the subcellular organelles and transporters present in tanycyte protrusions. 3V, third ventricle; ER, endoplasmic reticulum; MVB, multivesicular bodies.

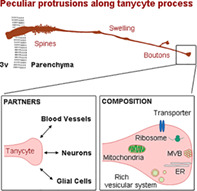

## 1 INTRODUCTION

The ependyma lining the ventricular system and the ependymal canal is largely described as a single layer of multiciliated and cuboidal cells (Jiménez, Domínguez‐Pinos, Guerra, Fernández‐Llebrez, & Pérez‐Fígares, 2014). Nevertheless, some midline structures around the third and fourth ventricles called circumventricular organs are lined by highly specialized ependymoglial cells called tanycytes (Langlet, Mullier, Bouret, Prevot, & Dehouck, 2013). Initially described along the lateral walls and the floor of the third ventricle (3V), tanycytes directly contact the cerebrospinal fluid through their apical surface, and send a single basal process that extends into hypothalamic regions including the median eminence (ME), the arcuate nucleus (ARH), the ventromedial nucleus (VMH) and the dorsomedial nucleus (DMH) (Mullier, Bouret, Prevot, & Dehouck, 2010). Since their characterization in 1954 by Horstmann (Horstmann, 1954), numerous studies clearly distinguished diverse tanycyte subpopulations along the 3V, so that four different subgroups are currently defined based on their morphological, structural, genetic and functional properties (Rodríguez et al., 2005). Basically, *β2* tanycytes line the ME, *β1* tanycytes line the lateral evaginations of the infundibular recess and the ventromedial ARH (vmARH), *α2* tanycytes line the area of the dorsomedial ARH (dmARH), and *α1* tanycytes line the VMH and DMH (Langlet, 2019; Mirzadeh et al., 2017; Rodríguez, Guerra, Peruzzo, & Blázquez, 2019; Rodríguez et al., 2005).

This heterogeneity among tanycyte populations allows them to participate in the regulation of numerous neuroendocrine functions—such as the control of energy balance, reproduction and seasonal adaptations—through their diverse cellular properties including blood/brain traffic controllers, metabolic sensors, modulators of cell function, and neural stem/progenitor cells (Langlet, 2014, 2019; Prevot et al., 2018). However, their interactions with different neural populations and their integration within different neuronal networks to ensure these regulations are still poorly described. The aim of this study is to define the neuroanatomical basis for tanycyte/neural cell interactions. Focusing on tanycytes lining the ARH, the VMH, and the DMH, we observed along their processes peculiar protrusions, of which we extensively characterized the morphometry, the partners and the composition. Altogether, our results allowed us to speculate about specific tanycyte‐to‐neural cell communications.

## 2 MATERIALS AND METHODS

### 2.1 Animals, and tdTomato expression in tanycytes

Two‐to‐four‐month‐old male Rosa26‐floxed stop tdTomato mice (*n* = 14, initially obtained from Charles River), two‐month‐old male Rosa26‐floxed stop tdTomato:NPY‐GFP mice (*n* = 4) and two‐month‐old male Rosa26‐floxed stop tdTomato:POMC‐GFP mice (*n* = 3) were used in this study. Animals were housed in groups (from 2 to 5 mice per cage), and maintained in a temperature‐controlled room (at 22–23°C) on a 12 hr light/dark cycle with ad libitum access to chow diet (Diet 3,436; Provimi Kliba AG, Kaiseraugst, Switzerland). All animal procedures were performed at the University of Lausanne, and were reviewed and approved by the Veterinary Office of Canton de Vaud. To induce tdTomato expression in tanycytes, TAT‐CRE fusion protein (Excellgen, EG‐1001) was stereotactically infused into the lateral ventricle (2 μl over 2 min at 2 mg/ml; at the coordinates from the bregma of anteroposterior = −0.3 mm; mediolateral = −1 mm and dorsoventral = −2.3 mm from the cortex surface) of ketamine/xylazine‐anesthetized mice (100 mg/kg and 20 mg/kg, respectively) 72 hr before experiments. This injection through the lateral ventricle avoids local inflammation around the 3V and sparsely label 3V tanycytes, optimizing their morphological analysis.

### 2.2 Tissue preparation

For immunostaining, mice were anesthetized with isoflurane, and perfused transcardially with 0.9% saline followed by an ice‐cold solution of 4% paraformaldehyde in 0.1 M phosphate buffer, pH 7.4. Brains were quickly removed, postfixed in the same fixative for 2 hr at 4°C, and immersed in 20% sucrose in 0.1 M phosphate buffered saline (PBS) at 4°C overnight. Brains were finally embedded in ice‐cold OCT medium (optimal cutting temperature embedding medium, Tissue Tek, Sakura) and frozen on dry ice or in liquid nitrogen‐cooled isopentane.

To visualize blood vessels using fluorescent dextran, mice were given i.v. injections of 70 kDa fluorescein isothiocyanate‐dextran (25 mg/ml, Cat Nb 90,718, lot # BCBV4422, Sigma, France) in sterile 0.9% saline (100 μl) into the tail vein and killed by decapitation 5 min later. Brains were then immersed in an ice‐cold solution of 4% paraformaldehyde in 0.1 M phosphate buffer, pH 7.4, for 24 hr at 4°C, followed by 20% sucrose in 0.1 M PBS at 4°C overnight. Brains were finally embedded in ice‐cold OCT medium and frozen on dry ice or in liquid nitrogen‐cooled isopentane.

For electron microscopy, mice were anesthetized with isoflurane, and perfused transcardially with 0.9% saline followed by an ice‐cold solution of 2% paraformaldehyde/2% glutaraldehyde in 0.1 M phosphate buffer, pH 7.4. Brains were quickly removed, postfixed in the same fixative overnight at 4°C. Two hundred micrometer‐thick hypothalamic slices were then cut using vibratome. TdTomato fluorescence in tanycytes was then acquired using ZEISS Axio Imager.M2 microscope equipped with ApoTome.2 in order to give coordinates to each protrusion in the slice. Afterwards, the samples were incubated in 2% (wt/vol) osmium tetroxide and 1.5% (wt/vol) K4[Fe(CN)6] in 0.1 M PB buffer for 1 hr, following by one‐hour incubation in 1% (wt/vol) tannic acid in 0.1 M PB buffer. Subsequently, brain slices were incubated in 1% (wt/vol) uranyl acetate for 1 hr and dehydrated at the end of standard gradual dehydration cycles in ethanol. Samples were flat embedded in Epon‐Araldite mix (Kolotuev, 2014; Kolotuev, Schwab, & Labouesse, 2009). All procedures were performed at room temperature.

### 2.3 Immunohistochemistry

Brains were cut using a cryostat into 20‐μm‐thick coronal, horizontal, or sagittal sections and processed for immunohistochemistry as described previously (Langlet, Mullier, et al., 2013). For most of the antibodies, slide‐mounted sections were (a) blocked for 30 min using a solution containing 4% normal goat serum and 0.3% Triton X‐100; (b) incubated overnight at 4°C with primary antibodies (Table 1) followed by 2 hr at room temperature with a cocktail of secondary Alexa Fluor‐conjugated antibodies (1:500, Molecular Probes, Invitrogen, San Diego, CA, Table 2); (c) counterstained with DAPI (1:10,000, Molecular Probes, Invitrogen), and (d) coverslipped using Mowiol (Calbiochem, La Jolla, CA). For HuC/D, slide‐mounted sections were first incubated in a boiling 10 mM Citrate Buffer solution, pH 6.0, for 12 min. For MCT1 immunostaining, 25‐μm‐thick free‐floating sections were 1) blocked for 30 minutes using a solution containing 2% normal donkey serum and 0.3% Triton X‐100; 2) incubated for 72 hr at 4°C with a primary rabbit polyclonal MCT1 antibody (1:500, characterized in Pierre, Pellerin, Debernardi, Riederer, & Magistretti, 2000) followed by 2 hr at room temperature with a secondary anti‐Rabbit FluoProbes^®^ 642 antibody (Interchim, FP‐DARBTTGX642).

**TABLE 1:** Primary antibodies used in the study

Antigen	Immunogen	Manufacturer	Cat number	Lot number	RRID	Species	Poly‐/monoclonal	Dilution	Pre‐treatment required
Vimentin (VIM)	Recombinant Golden Syrian hamster vimentin	Merck & Millipore	AB5733	2,987,458, 2,947,246, 3,215,975	AB_11212377	Chicken	Polyclonal	1:800	
ELAV‐like protein 3 and 4 (HuC/HuD)	Human HuC/HuD neuronal protein.	Invitrogen	16A11	1,963,099	AB_221448	Mouse	Monoclonal	1:200	Antigen retrieval with citrate buffer solution
Red fluorescent protein (RFP)	Red fluorescent protein (RFP) fusion protein corresponding to the full‐length amino acid sequence (234aa) derived from the mushroom polyp coral Discosoma.	Rockland	600–401‐379	35,634	AB_2209751	Rabbit	Polyclonal	1:1000	Antigen retrieval with citrate buffer solution
Neuron‐specific nuclear protein (NeuN)	Purified cell nuclei from mouse brain	Merck & Millipore	MAB377	2,919,676	AB_2298772	Mouse	Monoclonal	1:500	
Platelet endothelial cell adhesion molecule (CD31)	129/Sv mouse‐derived endothelioma cell line tEnd.1	BD biosciences	550,274	7,131,993	AB_393571	Mouse	Monoclonal	1:5000	
Glial fibrillary acidic protein (GFAP)	GFAP isolated from cow spinal cord	Agilent	Z0334	20,049,468	AB_10013382	Rabbit	Polyclonal	1:1000	
Transmembrane protein 119 (TMEM 119)	Recombinant protein corresponding to AA 189 to 280 from mouse TMEM119	Synaptic system	400,002	400,002/1–2	AB_2721104	Rabbit	Polyclonal	1:500	
Chondroitin sulfate proteoglycan (NG2)	Immunoaffinity purified NG2 chondroitin sulfate proteoglycan from rat.	Merck & Millipore	AB5320	3,118,137	AB_91789	Rabbit	Polyclonal	1:500	
Oligodendrocyte transcription factor 2 (OLIG2)	Synthetic peptide corresponding to AA 242 to 259 from mouse Olig2	Synaptic system	292,003	292,003/2	AB_2620030	Rabbit	Polyclonal	1:200	
Myelin basic protein (MBP)	Full length native protein (purified) corresponding to myelin basic protein aa 1 to the C‐terminus (database link: P02687)	Abcam	ab209328	GR3215004‐1	AB_2818988	Human	Monoclonal	1:200	
Solute carrier family 17 member 6 (VGLUT2)	Recombinant protein corresponding to AA 510 to 582 from rat VGLUT2 (UniProt id: Q9JI12)	Synaptic system	135,403	135,403/1–59	AB_887883	Rabbit	Polyclonal	1:500	
Solute carrier family 32 member 1 (VGAT)	Recombinant protein corresponding to AA 2 to 115 from rat VGAT (UniProt id: O35458)	Synaptic system	131,004	131,004/1–36	AB_887873	Guinea pig	Polyclonal	1:500	
Neurokinin B (NKB)	Pro‐NKB peptide coupled to human serum albumin by glutaraldehyde	Gift from P. Ciofi	IS‐39	‐	AB_2819032	Rabbit	Polyclonal	1:5000	
Tyrosine hydroxylase (TH)	Full length SDS denatured protein (purified from pheochromocytoma)	Abcam	ab112	GR131408‐18	AB_297840	Rabbit	Polyclonal	1:500	
Dynorphin B (DynB)	Dynorphin peptide coupled to human serum albumin by glutaraldehyde	Gift from P. Ciofi	IS‐35	‐	AB_2819033	Rabbit	Polyclonal	1:5000	
Pan actine (ACT)	Synthetic peptide within human beta Actin aa 1–100 (peptide available as ab28691, ab13772)	Abcam	ab8227	GR3212282‐1	AB_2305186	Rabbit	Polyclonal	1:500	
Translocase of outer mitochondrial membrane 20 (TOMM20)	Recombinant fragment within human TOMM20 aa 1 to the C‐terminus (database link: Q15388)	Abcam	ab186735	GR3228157‐1	AB_2716623	Rabbit	Monoclonal	1:250	
Phospho S6 ribosomal protein (pS6)	Synthetic phosphopeptide corresponding to residues surrounding Ser235 and Ser236 of human ribosomal protein S6	Cell signaling	#2211	23	AB_331679	Rabbit	Polyclonal	1:200	
Glutamate transporter 1 (GLT1)	Synthetic peptide from the carboxy‐terminus of rat GLT‐1.	Merck & Millipore	AB1783	2,987,435	AB_90949	Guinea pig	Polyclonal	1:500	
Solute carrier family 16 member 1 (MCT1)	Stnthetic peptide with 16 carboxyl‐terminal amino acids of Chinese hamster MCT1 (CPQQNSSGDPAEEESPV) and a cysteine added at the N‐terminal	L. Pellerin laboratory	‐	‐	AB_2815015	Rabbit	Polyclonal	1:500	Floating sections
Caveolin‐1 (CAV)	Synthetic peptide corresponding to residues surrounding Glu20 of human caveolin‐1	Cell signaling	#3238	3	AB_2072166	Rabbit	Monoclonal	1:250	
Clathrin heavy chain (CLTC)	Full length native protein (purified) corresponding to human Clathrin	Abcam	ab2731	GR3207735‐5	AB_303256	Mouse	Monoclonal	1:100	
CD9 molecule (CD9)	Synthetic peptide within human CD9 aa 200 to the C‐terminus. The exact sequence is proprietary. Database link: P21926	Abcam	ab92726	GR3252550‐2	AB_10561589	Rabbit	Monoclonal	1:500	

**TABLE 2:** Secondary antibodies used in the study

Secondary antibodies	Conjugated	Manufacturer	Cat number	RRID	Dilution
Goat anti‐mouse IgG (H + L) cross‐adsorbed secondary antibody	Alexa Fluor 647	Thermo Fisher	A21235	AB_141693	1:500
Goat anti‐rabbit IgG (H + L) highly cross‐adsorbed secondary antibody	Alexa Fluor 647	Thermo Fisher	A21245	AB_141775	1:500
Goat anti‐Guinea pig IgG (H + L) highly cross‐adsorbed secondary antibody	Alexa Fluor 647	Thermo Fisher	A21450	AB_141882	1:500
Donkey anti‐chicken IgG (H + L) AffiniPure secondary antibody	Alexa Fluor 647	Jackson ImmunoResearch	703–605‐155	AB_2340379	1:500
Alpaca anti‐human IgG (H + L) AffiniPure secondary antibody	Alexa Fluor 647	Jackson ImmunoResearch	609–605‐213	AB_2721861	1:500
Goat anti‐chicken IgY (H + L) secondary antibody	Alexa Fluor 488	Thermo Fisher	A11039	AB_142924	1:500
Goat anti‐mouse IgG (H + L) cross‐adsorbed secondary antibody	Alexa Fluor 488	Thermo Fisher	A11001	AB_2534069	1:500
Goat anti‐rabbit IgG (H + L) cross‐adsorbed secondary antibody	Alexa Fluor 488	Thermo Fisher	A11008	AB_143165	1:500
Donkey anti‐rabbit IgG (H + L) secondary antibody	FluoProbes® 642	Interchim	FP‐DARBTTGX642	AB_2827748	1:500
Goat anti‐rabbit IgG (H + L) cross‐adsorbed secondary antibody	Alexa Fluor 568	Thermo Fisher	A11011	AB_143157	1:500

### 2.4 Antibody characterization

All primary and secondary antibodies used are listed in Tables 1 and 2 respectively. These antibodies are in the Antibody Registry.

The chicken polyclonal antibody to VIM (Vimentin) (Millipore Cat# AB5733, RRID:AB_11212377) produced a pattern of staining associated to tanycytes, ependymal cells and endothelial cells, similar to that described elsewhere in the literature (Langlet et al., 2013; Langlet, Mullier, et al., 2013, 2010; Parkash et al., 2015). This expression profile replicates the pattern of mRNA expression determined by in situ hybridization in the adult mouse (Allen brain atlas).

The mouse monoclonal antibody to HuC/HuD (ELAV‐like protein 3 and ELAV‐like protein 4) (Invitrogen Cat# A21271, RRID:AB_221448) was prepared against human peptide QAQRFRLDNLN‐C‐KLH conjugate. The antiserum recognizes the Elav family members HuC, HuD, and Hel‐N1, which are all neuronal proteins. The antibody labeled neuronal cell nuclei and perikarya, similar to that described elsewhere in the literature (Caron, Sachot, Prevot, & Bouret, 2010).

The rabbit polyclonal antibody to RFP (Red fluorescent protein) (Rockland Cat# 600‐401‐379, RRID:AB_2209751) was prepared against RFP fusion protein corresponding to the full length amino acid sequence (234aa) derived from the mushroom polyp coral Discosoma. The antibody labeled cells expressing tdTomato.

The mouse monoclonal antibody to NeuN (Neuron‐specific nuclear protein) (Millipore Cat# MAB377, RRID:AB_2298772) produced a pattern of staining associated to neuronal cells, similar to that described elsewhere in the literature (Z. Liu & Martin, 2006). According to the manufacturer, this antibody recognizes the expected bands in the 46–48 kDa range and possibly another band at ~66 kDa on western blot.

The mouse monoclonal antibody to CD31 (Platelet Endothelial Cell Adhesion Molecule) (BD Biosciences Cat# 550274, RRID:AB_393571) produced a pattern of staining associated to endothelial cells, similar to that described elsewhere in the literature (Z. Liu & Martin, 2006). According to the manufacturer, this antibody inhibits a variety of in vitro and in vivo functions mediated by CD31.

The rabbit polyclonal antibody to GFAP (Glial Fibrillary Acidic Protein) (Agilent Cat# Z0334, RRID:AB_10013382) produced a pattern of staining associated to astrocytes, and some tanycytes lining the dorsal ARH, similar to that described elsewhere in the literature (Langlet, Mullier, et al., 2013). Specificity of this antibody in mouse brain was also confirmed by immunohistochemistry in GFAP knockout mice (Hanbury, Ling, Wuu, & Kordower, 2003).

The rabbit polyclonal antibody to TMEM119 (Transmembrane Protein 119) (Synaptic Systems Cat# 400002, RRID:AB_2721104) produced a pattern of staining associated to microglia, similar to that described elsewhere in the literature (Garcia‐Agudo et al., 2019).

The rabbit polyclonal antibody to NG2 (Chondroitin Sulfate Proteoglycan 4) (Millipore Cat# AB5320, RRID:AB_91789) produced a pattern of staining associated to NG2 oligodendrocyte progenitors, similar to that described elsewhere in the literature (Papay et al., 2006). This antibody stains the expected band at 250 kDa on western blots (Shi, Shu, Liang, Yuan, & Tang, 2016).

The rabbit polyclonal antibody to OLIG2 (Oligodendrocyte Transcription Factor 2) (Synaptic Systems Cat# 292003, RRID:AB_2620030) produced a pattern of staining associated to oligodendrocytes, similar to that described elsewhere in the literature (Chung, Guo, Jiang, Pleasure, & Deng, 2013).

The human monoclonal antibody to MBP (myelin basic protein) (Abcam Cat# ab209328, RRID: AB_2818988) produced a pattern of staining associated to mature and myelinating oligodendrocytes, similar to that described elsewhere in the literature (S.‐H. Chung et al., 2013).

The guinea pig polyclonal antibody to VGAT (Solute Carrier Family 32 Member 1) (Synaptic Systems Cat# 131004, RRID:AB_887873) targets the cytoplasmic domain of vesicular GABA transporter VGAT and produced a pattern of staining similar to that described elsewhere in the literature (Fekete et al., 2015). According to the manufacturer, this antibody recognizes the expected band at 60 kDa by western blot of synaptic vesicle fraction of rat brain.

The rabbit polyclonal antibody to VGLUT2 (Solute Carrier Family 17 Member 6) (Synaptic Systems Cat# 135403, RRID:AB_887883) targets the vesicular glutamate transporter 2 (VGLUT2) and produced a pattern of staining similar to that described elsewhere in the literature (Toyoshima et al., 2009). According to the manufacturer, this antibody recognizes the expected band at 62 kDa by western blot of synaptic vesicle fraction of rat brain.

The rabbit polyclonal antibody to NKB (Neurokinin B) (Ciofi P. laboratory, IS‐39, RRID: AB_2819032) was prepared against NKB precursor peptide‐2 coupled to human serum albumin by glutaraldehyde. This antibody was characterized previously (Ciofi, Leroy, & Tramu, 2006): its labeling is not abolished by preabsorption with other tachykinins (neurokinin A (NKA) and substance P (SP)). Its expression profile replicates the pattern of mRNA expression determined by in situ hybridization in the adult mouse (Allen brain atlas).

The rabbit polyclonal antibody to TH (Tyrosine Hydroxylase) (Abcam Cat# ab112, RRID:AB_297840) targets neurons expressing tyrosine hydroxylase including dopaminergic, noradrenergic and adrenergic neurons. It produced a pattern of staining similar to that described elsewhere in the literature (X. Liu et al., 2011).

The rabbit polyclonal antibody to DynB (Dynorphin B) (Ciofi P. laboratory, IS‐35, RRID: AB_2819033) was prepared against dynorphin B coupled to human serum albumin by glutaraldehyde. This antibody was characterized previously (Ciofi et al., 2006): its labeling is only abolished by preabsorption with the respective antigen. This expression profile replicates the pattern of mRNA expression determined by in situ hybridization in the adult mouse (Allen brain atlas).

The rabbit polyclonal antibody to ACT (pan‐actin) (Cell Signaling Technology Cat# 8456, RRID:AB_10998774) was validated by the manufacturer: it stains the expected band at 45 kDa on western blots of mouse brain.

The rabbit monoclonal antibody to TOMM20 (Translocase Of Outer Mitochondrial Membrane 20) (Abcam Cat# ab186734, RRID:AB_2716623) targets a mitochondrial marker, and produced a pattern of staining similar to that described elsewhere in the literature (Ioannou et al., 2019). This antibody was validated by flow cytometry and western blot by the manufacturer.

The rabbit polyclonal antibody to pS6 (phospho‐S6 Ribosomal protein) (Cell Signaling Technology Cat# 2211, RRID:AB_331679) was validated by the manufacturer: it stains the expected band at 32 kDa on western blots.

The rabbit polyclonal antibody to MCT1 (Solute Carrier Family 16 Member 1) (Pellerin L. laboratory, RRID:AB_2815015) was prepared against the carboxyl‐terminal amino acids of Chinese hamster MCT1 (CPQQNSSGDPAEEESPV). This antibody was characterized previously (Pierre et al., 2000). Briefly, peptide antigens were used as competitive inhibitors, by pre‐absorbing the MCT1 primary antibody with 10 μg/ml of the appropriate peptide antigen: staining was absent on membranes or in sections that had been incubated in such solutions (Pierre et al., 2000).

The guinea pig polyclonal antibody to GLT1 (Solute Carrier Family 1 Member 2) (Millipore Cat# AB1783, RRID:AB_90949) was evaluated by western blot of mouse brain membrane lysates: it stains the expected band at 62 kDa. Moreover, it produced a pattern of staining similar to that described elsewhere in the literature (Chung, Chen, Chan, & Yung, 2008).

The rabbit monoclonal antibody to CAV1 (Caveolin‐I) (Cell Signaling Technology Cat# 3238, RRID:AB_2072166) stains the expected band at 21 kDa on western blots of Hela Cell Extracts.

The mouse monoclonal antibody to CLTC (Clathrin Heavy Chain) (Abcam Cat# ab2731, RRID:AB_303256) detects clathrin heavy chain and stains the expected band at 190 kDa on western blots of bovine brain.

The rabbit monoclonal antibody to CD9 (CD9 molecule) (Abcam Cat# ab92726, RRID:AB_10561589) detects synthetic peptide within Human CD9 aa 200 to the C‐terminus. This antibody was validated by western blot of WT and KO A549 cell lysate: it stains the expected band at 25 kDa in WT cells but not in KO cells.

### 2.5 Microscopic imaging

Sections were analyzed using an ZEISS Axio Imager.M2 microscope, equipped with ApoTome.2 and a Camera Axiocam 702 mono (Zeiss, Germany). Specific filter cubes were used for the visualization of green (Filter set 38 HE eGFP shift free [E] EX BP 470/40, BS FT 495, EM BP 525/50), red (Filter set 43 HE Cy 3 shift free [E] EX BP 550/25, BS FT 570, EM BP 605/70), far red (Filter set 50 Cy 5 shift free [E] EX BP 640/30, BS FT 660, EM BP 690/50) and blue (Filter set 49 DAPI shift free [E] EX G 365, BS FT 395, EM BP 445/50) fluorescence. Different magnifications were selected using a Zeiss x20 objective (Objective Plan‐Apochromat ×20/0.8 M27 [FWD = 0.55 mm]), as well as a 63× oil‐immersion objective (Objective C Plan‐Apochromat ×63/1.4 Oil DIC M27 [FWD = 0.14 mm]). To create photomontages, images were acquired using ZEN 2.3 pro software using Z‐Stack and Tiles/Positions ZEN modules for each fluorophore sequentially. Quintuple‐ApoTome frames were collected in a stepwise fashion over a defined z‐focus range corresponding to all visible fluorescence within the section: basically, multiple‐plane frames were collected at a step of 0.3 μm while using x63 objective (between 35 and 45 frames per image) and 1 μm while using ×20 objective (between 4 and 10 frames per image). Weak deconvolution was finally applied on images following the acquisition. All images were then saved in .cvi, processed to get orthogonal and maximal intensity projections, and finally export in .tiff. For the processing steps (i.e., adjust brightness and contrast, change colors and merge images using Adobe Photoshop [Adobe Systems, San Jose, CA]).

For morphological analyses, images were acquired using Zeiss LSM 710 confocal microscope (Zeiss, Germany) with 561 nm laser and ZEN black 2012 software. High magnifications were obtained with a Plan‐Apochromat 20x W objective with a 1.0 NA (for the analysis of ependyma/tanycyte ratio, and nucleus area occupied by tanycyte processes) and an Apochromat ×63 Water immersion DIC objective with a 1.2 NA (for morphometric analysis of tanycyte protrusions). Multiple‐plane frames were collected at a step of 0.25 μm while using ×63 objective and 0.5 μm while using ×20 objective, over a defined z‐focus range corresponding to all visible fluorescence within the section. All images were then saved in .lsm for Imaris^®^ analysis.

### 2.6 Electron microscopy

The particularity of tanycyte endfeet analysis using electron microscopy is that they are rather large to be observed by a classical transmission electron microscopy (TEM) method for the volume acquisitions. Array tomography approach (Smith, 2018) consequently allowed us both to cover the large surface and make an efficient screening for the desired ROI. Moreover, as it is not a destructive technique such as Focused Ion Beam (FIB), it also permitted to go back to the area of interest and concentrate on desired details. To do so, polymerized flat blocks were trimmed using 90° diamond trim tool, and the arrays of 80 nm sections were obtained using 35° ATC diamond knife (Diatome, Biel, Switzerland) mounted on Leica UC6 microtome (Leica, Vienna). Sections were directly transferred to 2 × 4 cm pieces of silicon wafers using a modified array tomography procedure (Burel et al., 2018). During the sectioning phase, reliable landmarks were used to improve our chances to find tanycyte endfeet by applying a semi‐correlative approach: our regions of interest (ROI) were defined before cutting by superimposing the images of the fluorescent acquisition from the vibratome sections with the images of the embedded samples (Burel et al., 2018; Kolotuev, 2014; Kolotuev et al., 2009). Wafers were analyzed using FEI Helios Nanolab 650 scanning electron microscope (Thermo Fischer, Eindhoven). The imaging settings were as follows: MD detector, accelerating voltage 2 kV, current 0.8 nA, dwell time 4‐6 μs. Images were collected manually or using the AT module of MAPs program (Thermo Fischer, Eindhoven). Single images were aligned and reconstructed with the IMOD software package (Kremer, Mastronarde, & McIntosh, 1996). For electron microscopy data interpretation, previous reports in the literature were used to recognize the different neural cell types based on their ultrastructural characteristics (Luse, 1956).

### 2.7 Morphometric analysis

To quantitatively analyze tanycyte morphology and the subcellular protrusions observed along their process, three male mice were used. Three‐dimensional reconstructions of the image volumes were then prepared using Imaris^®^ visualization software to perform morphometric analysis. For ependyma/tanycyte ratio, the length of the ventricle occupied by tanycytes was reported to the total length of the ventricle: the length of the ventricle occupied by tanycytes (in μm) represents the distance from the bottom of the ventricle up to the last tanycyte measured using tdTomato fluorescence, whereas the total length of the ventricle (in μm) represents the distance from the bottom to the top of the ventricle measured using DAPI counterstaining. Three ratios per anteroposterior zone were used for quantification. For nucleus area occupied by tanycyte processes, the area of nucleus containing tanycyte processes was reported to the total area of the nucleus: the area of nucleus containing tanycyte processes (in μm^2^) represents the area within the nucleus of interest measured by delineating tdTomato fluorescence, whereas the total area of the nucleus (in μm^2^) represents the area of the nucleus of interest measured using DAPI counterstaining. Two ratios per anteroposterior zone and per nucleus were used for quantification. For cell body analysis, the maximal length of four cell bodies per anteroposterior zone and per nucleus were measured on an anteroposterior, ventrodorsal, and mediolateral direction. For process thickness, the maximal diameter of four processes per anteroposterior zone and per nucleus was measured at the proximal, medial, and distal portion of the process. For protrusion analysis, each protrusion was first defined as *Surface* using Imaris^®^ software, and their surface area, volume and sphericity were then quantified using Imaris^®^ algorithm.

### 2.8 Tanycyte partner analysis

To quantitatively analyze the proportion of tanycyte interactions with different neural cells, we first counted the number of tanycyte protrusions (e.g., swelling and boutons) in the region of interest, and then the number of these protrusions in contact with a neural partner. The cell identity of these partners was assessed by immunohistochemistry for known markers (i.e., HuC/HuD for neuronal cells, CD31 for vessels, GFAP for astrocytes, NG2 and MBP for oligodendrocytes, and TMEM19 for microglia), while NPY and POMC were visualized using transgenic reporter mice. The regions of interest (i.e., ARH, VMH, and DMH) were identified based on DAPI staining. The analysis was performed in three mice per staining, in 2 sections per anteroposterior zone.

### 2.9 Statistical analysis

All values are expressed as means ± *SEM*. Data were analyzed for statistical significance with Graph Prism 5 software (Version 11.0), using one‐way analysis of variance (ANOVA) followed by a Tukey's post hoc test or two‐way ANOVA followed by a Bonferroni's post hoc test when appropriate. *p*‐values of less than .05 were considered to be statistically significant.

## 3 RESULTS

### 3.1 Location and direction of tanycyte processes within the hypothalamic parenchyma

**FIGURE 1:**
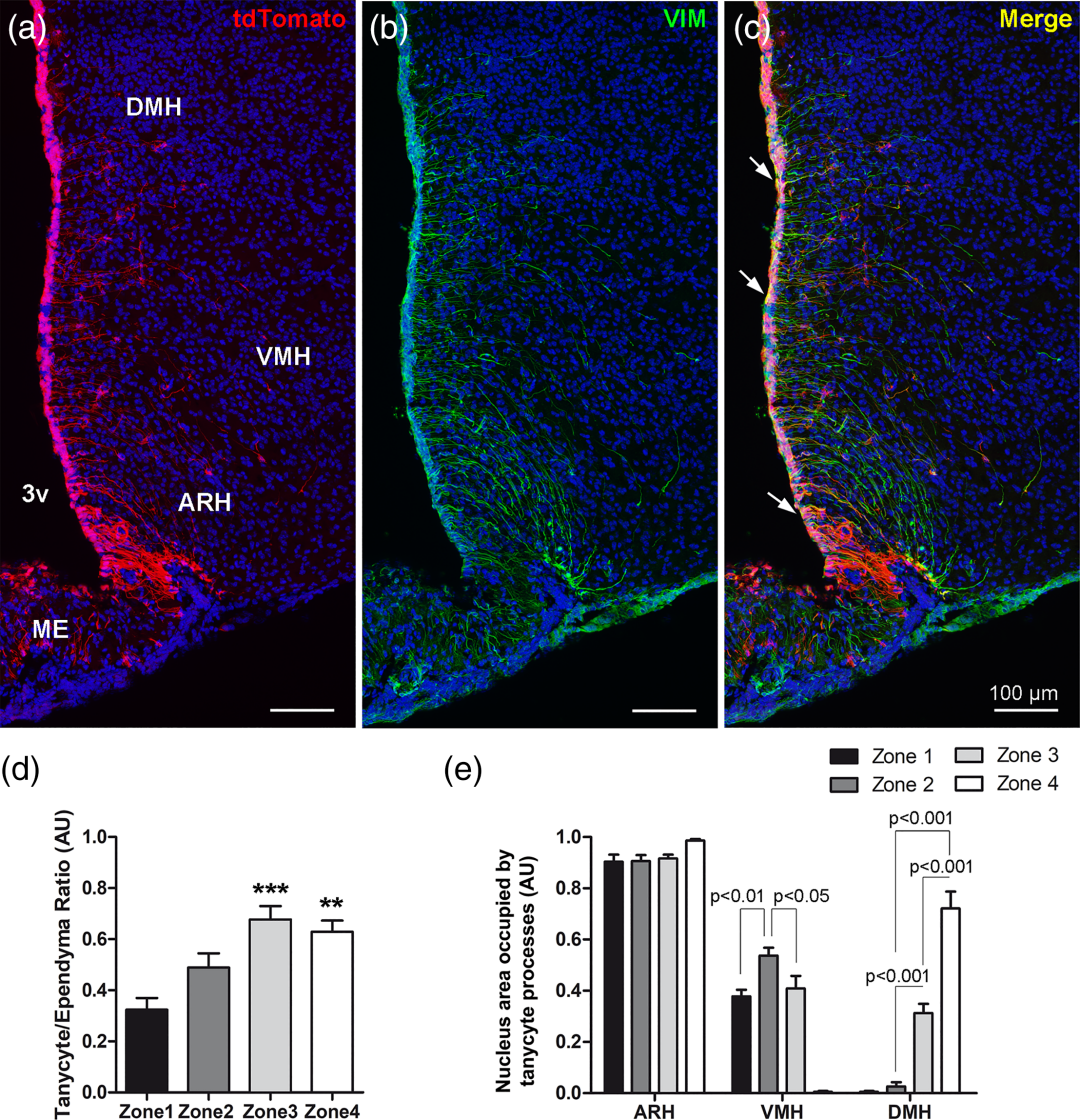
Hypothalamic nuclei targeted by tanycyte processes. (a–c) low‐magnification z‐stack images (×20) showing the distribution of tdTomato (red) (a), vimentin immunoreactivity (VIM, green) (b), and merge (c) with DAPI counterstaining (blue) in coronal section at bregma −1.9 mm (Zone 3). Vimentin immunoreactivity is distributed throughout the cells lining the third ventricle (b) and colocalizes with tdTomato (arrows in c). (d) Proportion of third ventricle formed by tanycytes per zone on the anteroposterior axis. (e) Proportion of ARH, VMH, and DMH nuclei containing tanycyte processes per zone on the anteroposterior axis. 3 V, third ventricle; ARH, arcuate nucleus; DMH, dorsomedial nucleus; ME, median eminence; VMH, ventromedial nucleus. Scale bars = 100 μm in a–c. Error bars represent *SEM* in (d,e). ***p < .001; **p < .01. Cf Figure S1 for experimental procedures [Color figure can be viewed at wileyonlinelibrary.com]

To examine the morphology of tanycytes lining the lateral wall of the 3V (commonly called dorsal β1 and α‐tanycytes), we filled their cytoplasm with the red fluorescent tdTomato protein using a transgenic approach. To do so, TAT‐CRE fusion protein was stereotactically infused into the lateral ventricle of *tdTomato* ^loxP/+^ cre reporter mice to induce tdTomato expression in ependymoglial cells, including tanycytes, as described previously (Langlet, Levin, et al., 2013; Parkash et al., 2015) and confirmed by vimentin immunostaining (VIM, Figure 1a–c). As TAT‐CRE mainly incorporates into cells located close to the site of injection (Langlet, Levin, et al., 2013), this injection in the lateral ventricle allowed us to sparsely label 3V tanycytes (Figure 1a–c), facilitating their morphological analysis. Using this approach, we confirm that 3V hypothalamic tanycytes are present from Bregma −1.3 to −2.5 mm in adult male mice and send their processes into the arcuate nucleus (ARH), the ventromedial (VMH) and the dorsomedial nucleus (DMH) (Figure 1a–c).

As the spatial distribution of neuronal populations within hypothalamic nuclei may be different on the ventrodorsal and anteroposterior axes, a systematic analysis was then performed to determine which nucleus subdivisions receive tanycyte processes in order to later unravel which neuronal populations likely interact with tanycytes. For this analysis, the region was divided in three subregions on the ventrodorsal axis, corresponding to the nuclei in which tanycyte processes are sent (namely the ARH, the VMH vs. the DMH); and in four subregions on the anteroposterior axis, corresponding to Zone 1 (from bregma −1.3 to −1.6 mm), Zone 2 (from bregma −1.6 to −1.8 mm), Zone 3 (from bregma −1.8 to −2.1 mm) and Zone 4 (from bregma −2.1 to −2.5 mm) (Figure S1). From a neuroanatomical point of view, these subdivisions were defined based on the shape of the ventricle, and the presence/absence of hypothalamic nuclei along the 3 V (Figure S1). For some analyses, ARH was also divided into ventromedial ARH (vmARH) versus dorsomedial ARH (dmARH). This anteroposterior and ventrodorsal analysis first shows that tanycytes are mainly located at the bottom of the 3V and that the tanycyte/ependymal cell ratio progressively grows from the rostral to the caudal region of the brain reaching up to 60% of the ventricular wall occupied by tanycytes in Zones 3 and 4 (Figure 1d). Secondly, we show that the entire ARH typically contains tanycyte processes, whereas only subdivisions of the VMH and DMH do so (Figure 1e): indeed, 40% of the VMH in Zones 1, 2, and 3, as well as up to 60% of the DMH in Zone 4 contain tanycyte processes (Figure 1e), corresponding respectively to the dorsomedial and central VMH, and the compact DMH.

**FIGURE 2:**
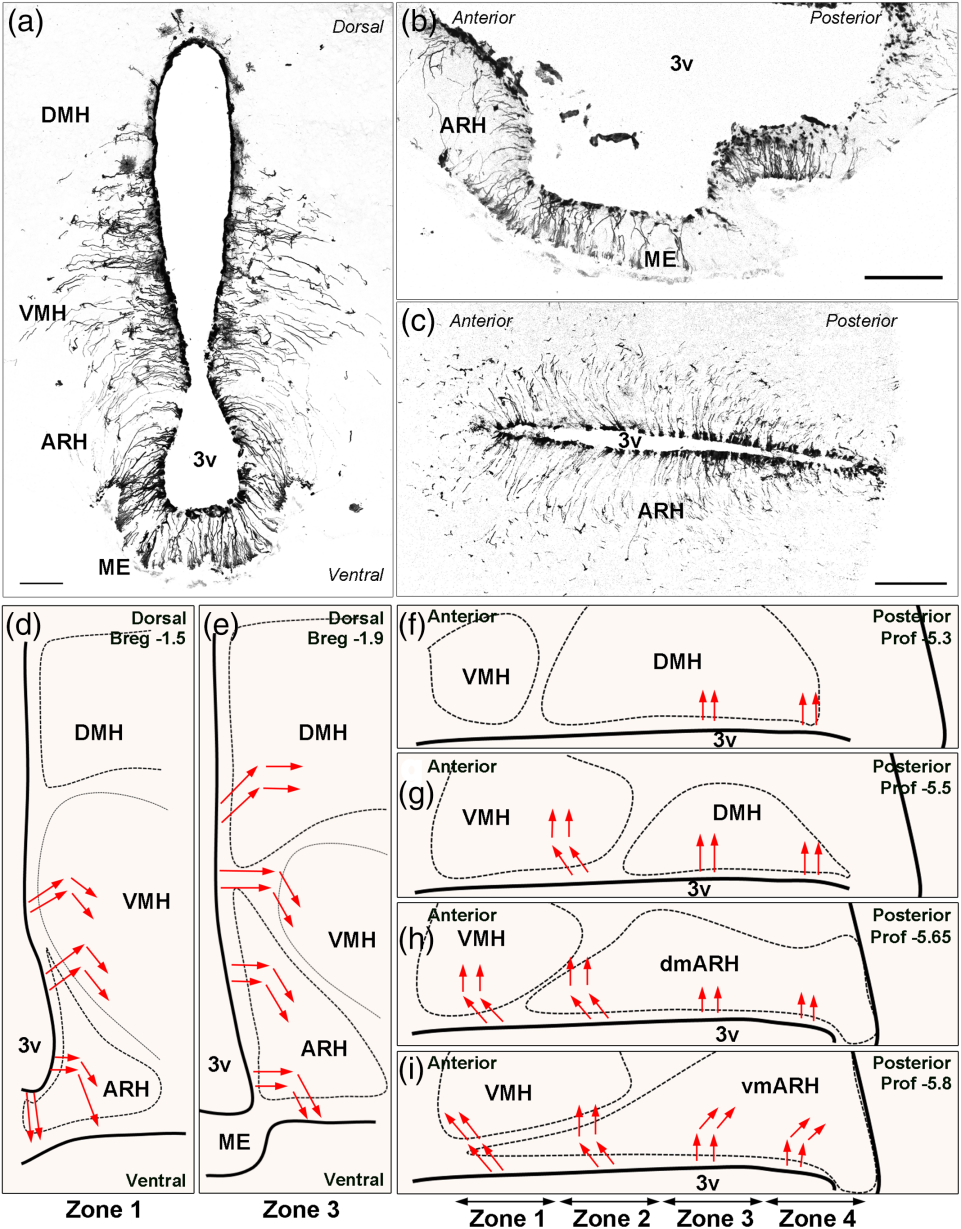
Trajectory of tanycyte processes through the hypothalamic parenchyma. (a–c) low‐magnification z‐stack images (×20) showing the distribution of tdTomato (black) in coronal (Bregma −1.7; thickness = 7 μm) (a), sagittal (midsagittal; thickness = 10 μm) (b), and horizontal (prof. ‐5.8; thickness = 14.3 μm) (c) sections. (d–i) Representation of tanycyte process trajectories (red arrows) in coronal (d–e) and horizontal sections (f–i). On coronal sections, trajectories of tanycyte processes in Zones 2 and 4 are similar to Zone 3. 3V, third ventricle; ARH, arcuate nucleus; DMH, dorsomedial nucleus; ME, median eminence; VMH, ventromedial nucleus. Scale bars = 100 μm in a–c [Color figure can be viewed at wileyonlinelibrary.com]

Using coronal, sagittal, and horizontal sections (Figure 2a–c, respectively), we finally determined the trajectory of tanycyte processes through the hypothalamic parenchyma (Figure 2d–i). Tanycytes lining the vmARH laterally project their processes in a rostral trajectory followed by a ventro‐rostral trajectory in Zone 1 (Figure 2d,i); in a rostral trajectory followed by a ventral trajectory in Zone 2 (Figure 2e,i); whereas they curve laterally and then follow a ventro‐caudal trajectory in Zones 3–4 (Figure 2e,i). Tanycytes lining the dmARH laterally project their processes in a dorso‐rostral trajectory followed by a ventral trajectory in Zone 1 (Figure 2d,h); in a rostral trajectory followed by a ventral trajectory in Zone 2 (Figure 2e,h); whereas they curve laterally and then ventrally in Zones 3–4 (Figure 2e,h). Tanycytes lining the VMH laterally project their processes into the brain parenchyma in a dorso‐rostral trajectory followed by a ventral trajectory in Zone 1 (Figure 2d,h), in a rostral trajectory followed by a ventral trajectory in Zone 2 (Figure 2e,g), and in a lateral trajectory followed by a ventral trajectory in Zone 3 (Figure 2e,g). Tanycytes lining the DMH project their processes into the brain parenchyma following a dorsolateral trajectory followed by a lateral trajectory in Zones 3–4 (Figure 2e–g).

### 3.2 Presence of subcellular protrusions along tanycyte processes

**FIGURE 3:**
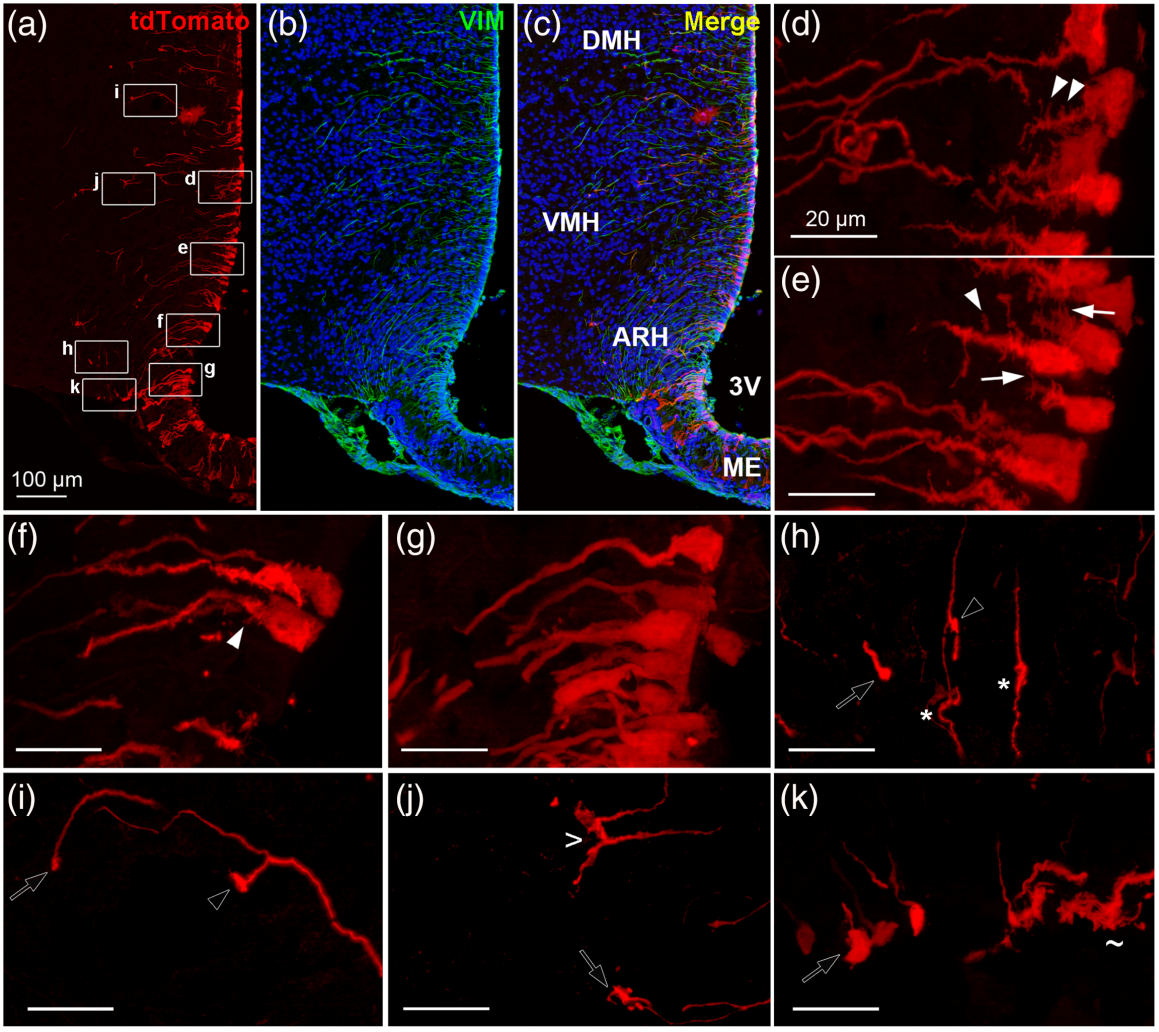
Different protrusions are observed along tanycyte processes lining the lateral wall of the third ventricle. (a–c) Low‐magnification z‐stack images (×20) showing the distribution of tdTomato (red) (a), vimentin immunoreactivity (VIM, green) with DAPI counterstaining (blue) (b), and merge (c) in coronal section in Zone 2 (Bregma −1.7). (d–k) High‐magnification z‐stack images (×63) of protrusions observed in tanycyte processes lining the lateral wall of the 3V, including spines (arrowheads in d–f) and spine contacts (arrows in e) within proximal processes; swelling (stars in h) and en passant boutons (empty arrowhead in h,i) along the process; and boutons (empty arrows in h–k), sleeves (> in j) and fork‐like endfeet (~ in k). Pictures (d–k) are the maximal intensity projections of z‐stack acquisition (thickness = 14.1 μm in d, 9.3 μm in e, 19.7 μm in f, 9.9 μm in g, 13.8 μm in h, 4.8 μm in i, 13.5 μm in j, 9 μm in k). 3V, third ventricle; ARH, arcuate nucleus; DMH, dorsomedial nucleus; ME, median eminence; VMH, ventromedial nucleus. Scale bars = 100 μm in a–c; 20 μm in d‐k. Cf Video S1 [Color figure can be viewed at wileyonlinelibrary.com]

**FIGURE 4:**
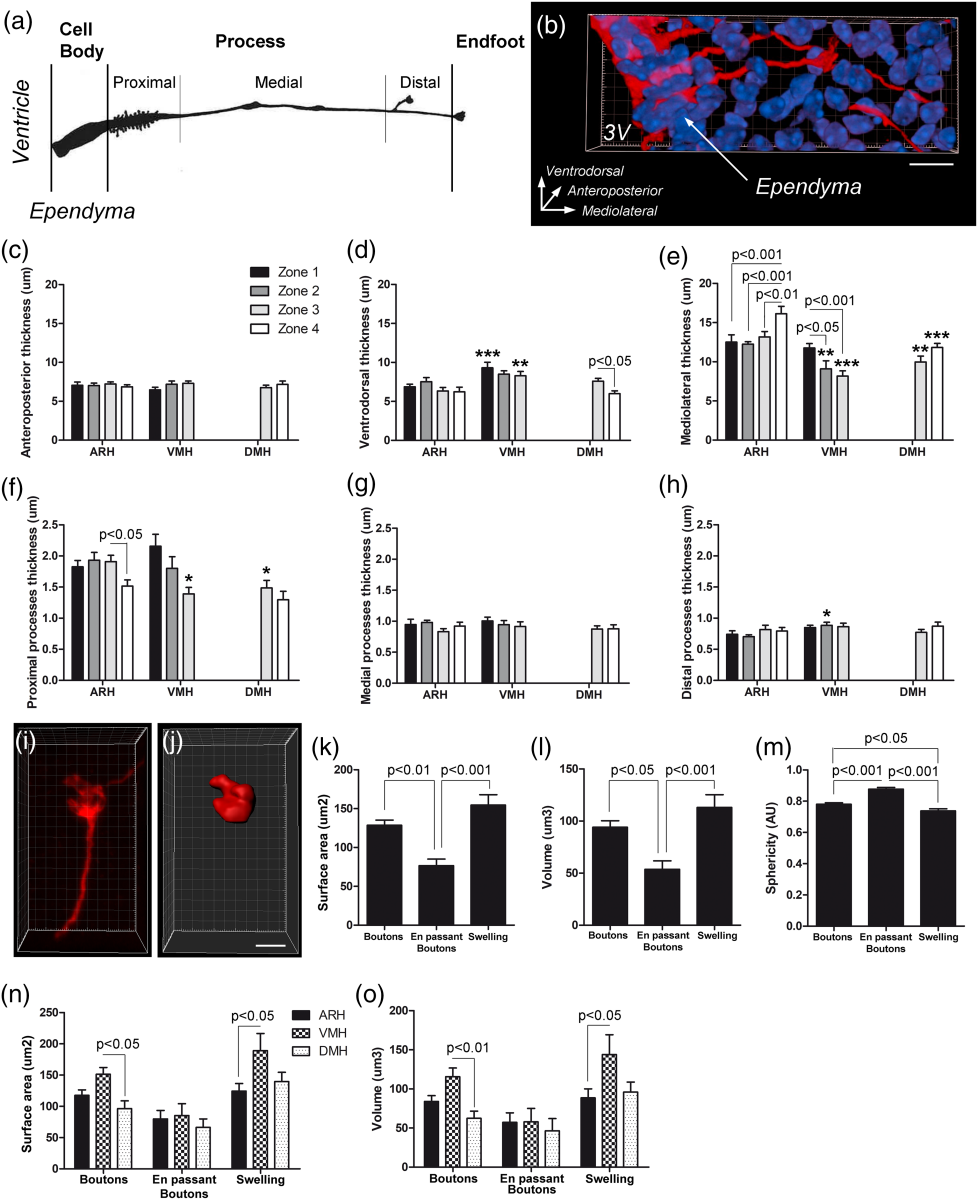
Morphometric analysis of different tanycyte subgroups. (a,b) Schematic representation (a) and Imaris® blend mode view of tanycyte (red) in the three‐dimension space (b) used for morphometric analysis. (c–e) Summary morphometric data of tanycyte cell body thickness collected using Imaris^®^ software per zone and per nuclei on the anteroposterior (c), the ventrodorsal (d) and the mediolateral axis (e). (f–h) Summary morphometric data of tanycyte process diameter collected using Imaris® software per zone and per nuclei. Diameter was measured at the proximal (f), medial (g), and distal (h) portion of the process. (i,j) High‐magnification z‐stack images (×63) of tdTomato fluorescence (i) and the associated image after Imaris® surface algorithm (j) used for protrusion morphometric analysis. (k–o) Surface area (k and n), volume (l and o), and sphericity (m) of tanycyte protrusions ‐including boutons, en passant boutons and swelling‐ collected using Imaris^®^ software on the entire region (k–m) and per nuclei (n,o). Scale bar = 2 μm in i,j. Error bars represent *SEM*. ***p < .001; **p < .01; *p < .05. Cf Figure S1 for experimental procedures [Color figure can be viewed at wileyonlinelibrary.com]

TdTomato fluorescent protein fills the entire cytoplasm of the cell: our approach consequently allowed us to study cell morphology in detail (Figure 3a–c). Tanycytes lining the lateral wall of the third ventricle share a similar shape composed of a somatic region, a long process and an endfoot (Figures 3a, 4a). The process may be additionally subdivided in three portions (Figures 3a, 4a): a proximal “neck” portion tapered from the cell body, a thin medial portion, and a thinner distal portion ending with an endfoot. Along this process, numerous peculiar protrusions—undetectable with vimentin immunostaining—were observed using our approach (Figure 3d–k). The neck region extends in the periventricular layer over about 20 μm and is characterized by the presence of spine‐like protrusions along the VMH and DMH—commonly named *α1* tanycytes—(Figure 3d), whereas this feature progressively disappears in dmARH tanycytes—commonly named *α2* tanycytes—(Figure 3e,f), while being absent in vmARH tanycytes—commonly named dorsal *β1* tanycytes—(Figure 3g). Interestingly, some of these spines connect to each other between neighboring tanycytes and/or other ependymoglial cells (Figure 3e, Video S1), suggesting the formation of close contacts between tanycyte processes. The medial and distal portions of dmARH, VMH and DMH tanycyte processes are tortuous with irregular swellings (Figure 3h) giving them a beaded aspect. The distal portion occasionally forms en passant boutons (Figure 3h,i), and finally ends making diverse formations including boutons (Figure 3h,i), claws (Figure 3j) or sleeves (Figure 3j). In contrast, vmARH tanycyte processes are quite smooth, and end at the pial surface forming club‐shaped endfeet laterally or fork‐shaped endfeet more medially (Figures 3k and S1).

### 3.3 Morphometric analysis of different tanycyte subtypes along the ventrodorsal and anteroposterior axes

To categorize tanycyte subgroups with specific morphometric features, morphometric analysis of tanycytes was then performed according to their location on both the ventrodorsal and anteroposterior axes (Figures 4 and S1).

First, we measured tanycyte cell body size using the Imaris^®^ software (Figure 4a–e). The mean thickness of the tanycyte cell body along the anteroposterior axis (Figure 4c) is similar in all nuclei and zones (mean size = 6.9 μm ±0.2). In contrast, their thickness along the ventrodorsal and mediolateral axes displays subtle differences (Figure 4d,e): indeed, the thickness of tanycyte cell body along the ventrodorsal axis is higher throughout the VMH (mean size = 9.2 μm ±0.3) compared with the ARH (mean size = 6.7 μm ±0.7) and the DMH (mean size = 6.6 μm ±0.6) (Figure 4d), whereas the thickness along the mediolateral axis (Figure 4e) is larger throughout the ARH (mean size = 13.5 μm ±0.7) compared with the DMH (mean size = 11.1 μm ±0.7) and the VMH (mean size = 9.1 μm ±0.9). In other words, the cell bodies of ARH and DMH tanycytes are typically elongated with their long axis at right angle to the ventricular surface, whereas VMH tanycytes are more flattened along the ventricle. These features are more pronounced in the posterior region. No difference was observed between vmARH and dmARH tanycyte cell bodies (data not shown).

Then, the proximal, medial, and distal portions of tanycyte processes were analyzed along the ventrodorsal and anteroposterior axes (Figure 4a, f–h). Tanycyte processes possess a rather uniform diameter going from 1.7 μm ±0.1 at the proximal neck portion, to 0.9 μm ±0.02 at the medial portion and finishing at 0.8 μm ±0.04 at the distal segment. Differences along the ventrodorsal and anteroposterior axes were nevertheless observed for the proximal neck portion: tanycyte processes are thinner in the caudal and dorsal part of the third ventricle (Figure 4f). The difference about the proximal neck portion also concerns the presence or absence of spines in the VMH/DMH and ARH (Figure 3d–g), respectively. When present, their mean length is 1.5 μm ±0.3 and does not differ along the ventrodorsal and anteroposterior axes.

Finally, some subcellular protrusions—namely swelling, en passant boutons and endfeet boutons—were morphologically analyzed using the *Surface* algorithm in Imaris^®^ software (Figure 4i,j): this algorithm allowed us to measure their surface area (Figure 4k,n), their volume (Figure 4l,o) and their sphericity (Figure 4m). While swellings and terminal boutons share the same surface area and volume, en passant boutons are smaller (Figure 4k,l). Indeed, the mean size of swellings is 3.4 × 5.6 × 9.4 μm (±1.1 × 1.3 × 3.7 respectively) and the one of boutons is 3.5 × 5.4 × 7.7 μm (±1.3 × 1.6 × 2.7, respectively). In contrast, the mean size of en passant boutons is 3.6 × 4.3 × 5.2 μm (±1.2 × 1.3 × 1.3, respectively). Moreover, en passant boutons are quite spherical whereas boutons and swellings are oval (Figure 4m). No difference was observed along the anteroposterior axis (data not shown). However, it is worth noting that terminal boutons and swellings are bigger in the VMH (Figure 4n–o).

### 3.4 Tanycyte protrusions in close proximity to different neural cells

**FIGURE 5:**
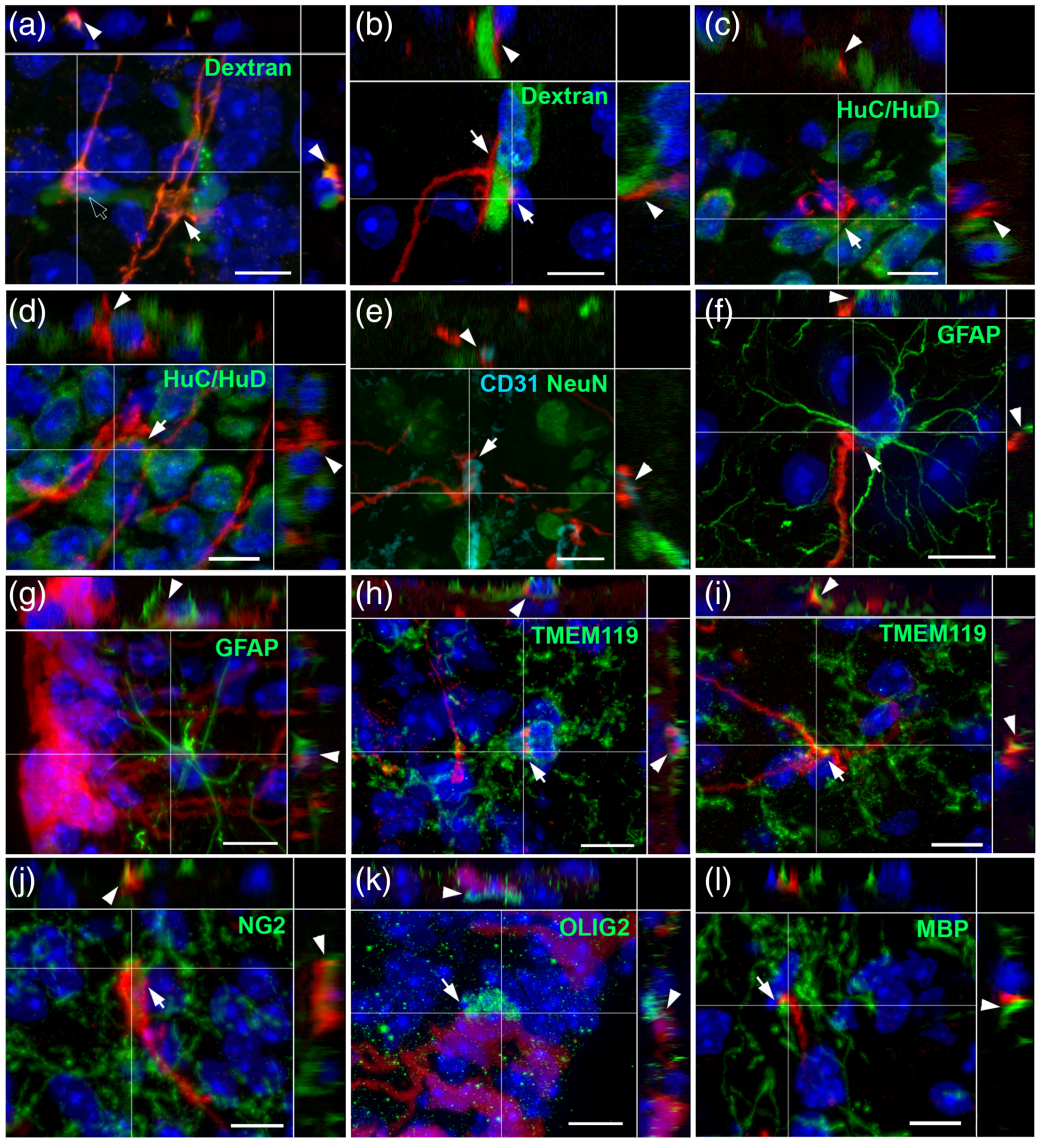
Tanycyte protrusions are in close proximity to different neural cells. (a–l) High‐magnification z‐stack images (×63) showing the distribution of tdTomato (red, a–l), and dextran fluorescence (green, a,b), HuC/HuD immunoreactivity (green, c,d), CD31 and NeuN immunoreactivity (cyan and green respectively, e), GFAP immunoreactivity (green, f–g), TMEM119 immunoreactivity (green, h,i), NG2 immunoreactivity (green, j), OLIG2 immunoreactivity (green, k), and myelin basic protein (MBP) immunoreactivity (green, l) with DAPI counterstaining (blue, a–d and f–l) in coronal sections in the arcuate nucleus, in Zone 2. Pictures (a–l) are the maximal intensity projections of z‐stack acquisition. Inset on the top in a–l panels shows the orthogonal view on the horizontal line; inset on the right in a–l panels shows the orthogonal view on the vertical line. Arrows and arrowheads indicate the site of contact in the coronal sections and in the orthogonal views, respectively. Low magnifications images are available upon request. Scale bars = 10 μm in a–l [Color figure can be viewed at wileyonlinelibrary.com]

We next analyzed which type of neural cells these protrusions—in particular sleeves, boutons, swellings and spines—are in contact with by using fluorescent dye and immunohistochemistry (Figure 5, Table 3).

**TABLE 3:** Proportion of tanycyte protrusions in contact with a neural partner along the anteroposterior axis (see Figure S1)

	Contacting protrusions/Total protrusions (percentage)				
	Zone 1	Zone 2	Zone 3	Zone 4	All zones
ARH vessels	19/28 (67.9%)	17/24 (70.8%)	14/21 (66.7%)	20/25 (80%)	70/98 (71.4%)
ARH neurons	8/29 (27.6%)	11/31 (35.5%)	9/26 (34.6%)	9/29 (31.0%)	37/115 (32.2%)
VMH neurons	7/27 (25.9%)	8/30 (26.7%)	9/24 (37.5%)	NA	24/81 (29.6%)
DMH neurons	NA	NA	11/34 (32.4%)	11/32 (34.4%)	22/66 (33.3%)
NPY neurons	18/65 (27.7%)	22/90 (24.4%)	16/80 (20.0%)	23/96 (24.0%)	79/331 (23.9%)
POMC neurons	10/100 (10.0%)	14/105 (13.3%)	10/119 (8.4%)	11/103 (10.7%)	45/427 (10.5%)
ARH astrocytic fibers	36/65 (55.4%)	39/58 (67.2%)	28/53 (52.8%)	24/47 (51.1%)	127/223 (57.0%)
ARH astrocyte cell bodies	6/65 (9.2%)	4/58 (6.9%)	8/53 (15.1%)	7/47 (14.9%)	25/223 (11.2%)
ARH NG2‐positive fibers	31/42 (73.8%)	29/38 (76.3%)	25/35 (71.4%)	34/50 (68.0%)	119/165 (72.1%)
ARH NG2‐positive cell bodies	2/42 (4.8%)	4/38 (10.5%)	6/35 (17.1%)	4/50 (8.0%)	16/165 (9.7%)
ARH myelinated fibers	3/14 (21.4%)	4/14 (28.6%)	4/19 (21.1%)	5/21 (23.8%)	16/68 (23.5%)
ARH microglial fibers	16/44 (36.4%)	18/38 (47.4%)	14/34 (41.2%)	14/39 (35.9%)	62/155 (40.0%)
ARH microglial cell bodies	4/44 (9.1%)	5/38 (13.2%)	7/34 (20.6%)	9/39 (23.1%)	25/155 (16.1%)

*Note:* Vessels were visualized by CD31 immunostaining, neurons by HuC/HuD immunostaining, astrocytes by GFAP immunostaining, NG2‐positive cells by NG2 immunostaining, myelinated fibers by MBP immunostaining, and microglia by TMEM119 immunostaining. NPY and POMC neurons were visualized using genetic mice model. This analysis was done in three mice, except for MBP immunostaining (*n* = 1). Abbreviations: ARH, arcuate nucleus; DMH, dorsomedial nucleus; NA, non applicable; VMH, ventromedial nucleus.

As previously described, the most frequently identified tanycyte partners are blood vessels visualized using i.v. injected fluorescent dextran (Figure 5a,b) or anti‐CD31 antibodies (Figure 5e) (up to 71% association; Table 3). These associations occur through two different tanycyte protrusions: sleeve‐like shapes formed by numerous tanycyte endfeet around the blood vessel, and boutons (arrows and empty arrows respectively, Figure 5a,b). In some cases, in particular in the vmARH, tanycyte processes were also observed surrounding a blood vessel before continuing their way into the brain parenchyma (data not shown).

Neurons visualized by HuC/HuD (Figure 5c,d) or NeuN (Figure 5e) immunostaining constitute other tanycyte partners: up to 33% of tanycyte boutons (Figure 5d,e) as well as swellings (Figure 5c) were observed in contact with soma (Table 3). In some cases, tanycyte endfeet separate neurons from blood vessels (Figure 5e).

Besides capillaries and neurons, associations with other neural cell types were also detected (Table 3). In particular, tanycytes contact astrocytes visualized by GFAP immunostaining: some tanycyte boutons end on the astrocyte cell body (Figure 5f), whereas astrocyte processes also appear to contact numerous neighboring tanycyte processes especially at the proximal neck portion (Figure 5g). Tanycytes also contact microglia, pre‐oligodendrocytes, immature oligodendrocytes and myelinating oligodendrocytes visualized by targeting TMEM119 (Figure 5h,i), NG2 (Figure 5j), OLIG2 (Figure 5k) and MBP (Figure 5l), respectively. Interestingly, tanycyte boutons end on the cell body of these cells (Figure 5h and k, Table 3) but are also wrapped by their processes (Figure 5i,j, and l, Table 3).

**FIGURE 6:**
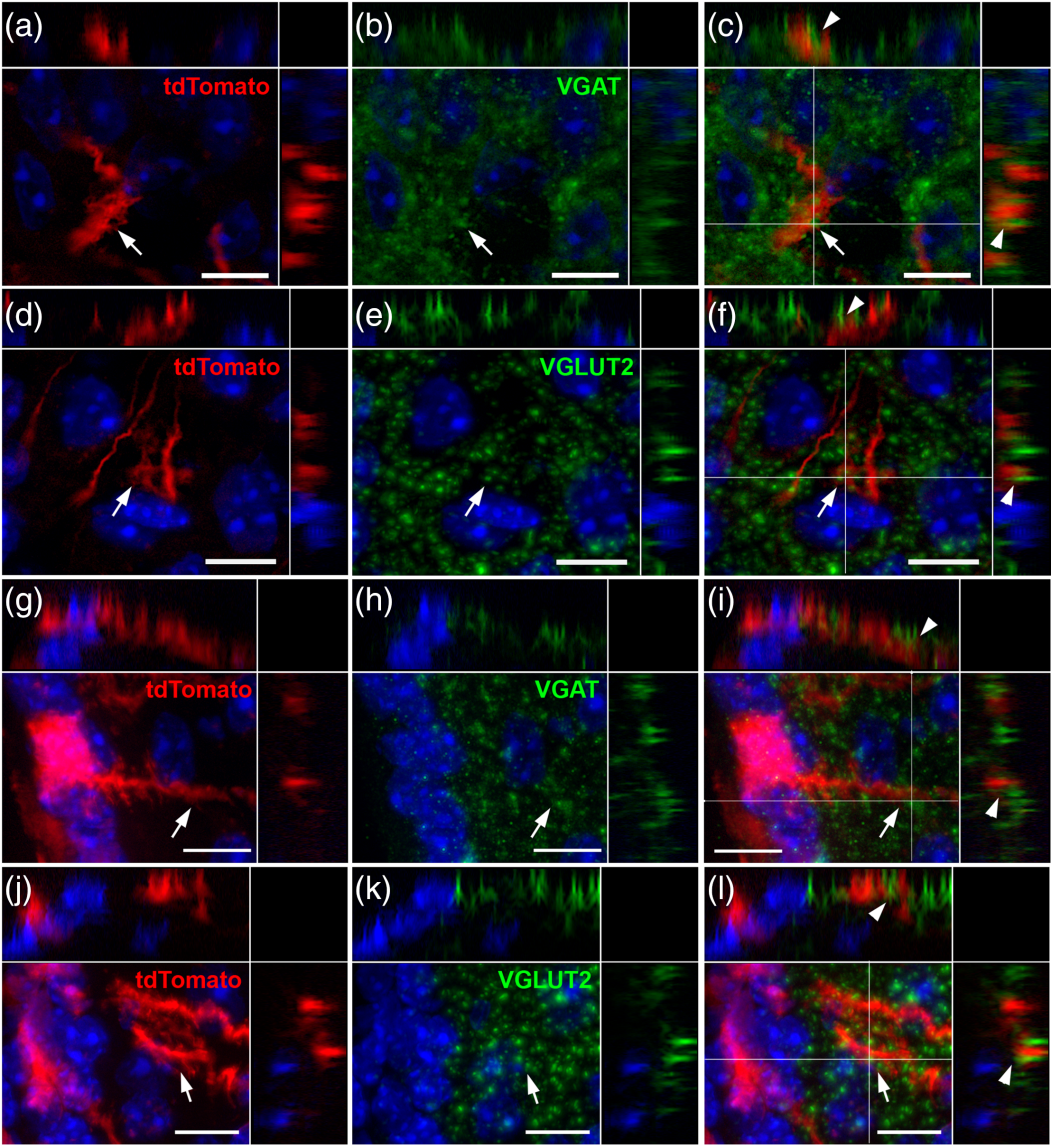
Tanycyte protrusions are in close proximity to synaptic terminals. (a–l) High‐magnification z‐stack images (×63) showing the distribution of tdTomato (red, a, d, g, and j), VGAT (green, b and h), VGLUT2 (green in e and k) immunoreactivity, and merge (c, f, i, and l) with DAPI counterstaining (blue, a–l). Images were acquired in coronal sections of the arcuate nucleus, in Zone 2. Pictures (a–l) are the maximal intensity projections of z‐stack acquisition. Inset on the top in a–l panels shows the orthogonal view on the horizontal line; inset on the right in a–l panels shows the orthogonal view on the vertical line. Arrows and arrowheads indicate the site of contact in the coronal sections and in the orthogonal views, respectively. Low magnifications images are available upon request. Scale bars = 10 μm in a–l [Color figure can be viewed at wileyonlinelibrary.com]

Finally, tanycyte associations with glutamatergic and GABAergic terminals were analyzed using VGLUT2 and VGAT immunostainings, respectively (Figure 6). Along tanycyte processes, swellings and boutons wrap GABAergic (Figure 6a,c) and, to a lesser extent, glutamatergic terminals in the ARH (Figure 6d–f). In contrast, at the proximal neck portion along the VMH and DMH, GABAergic and glutamatergic terminals end on tanycyte spines (Figure 6g–i and j–l, respectively).

### 3.5 Tanycyte terminal boutons contact diverse arcuate neuronal subpopulations

**FIGURE 7:**
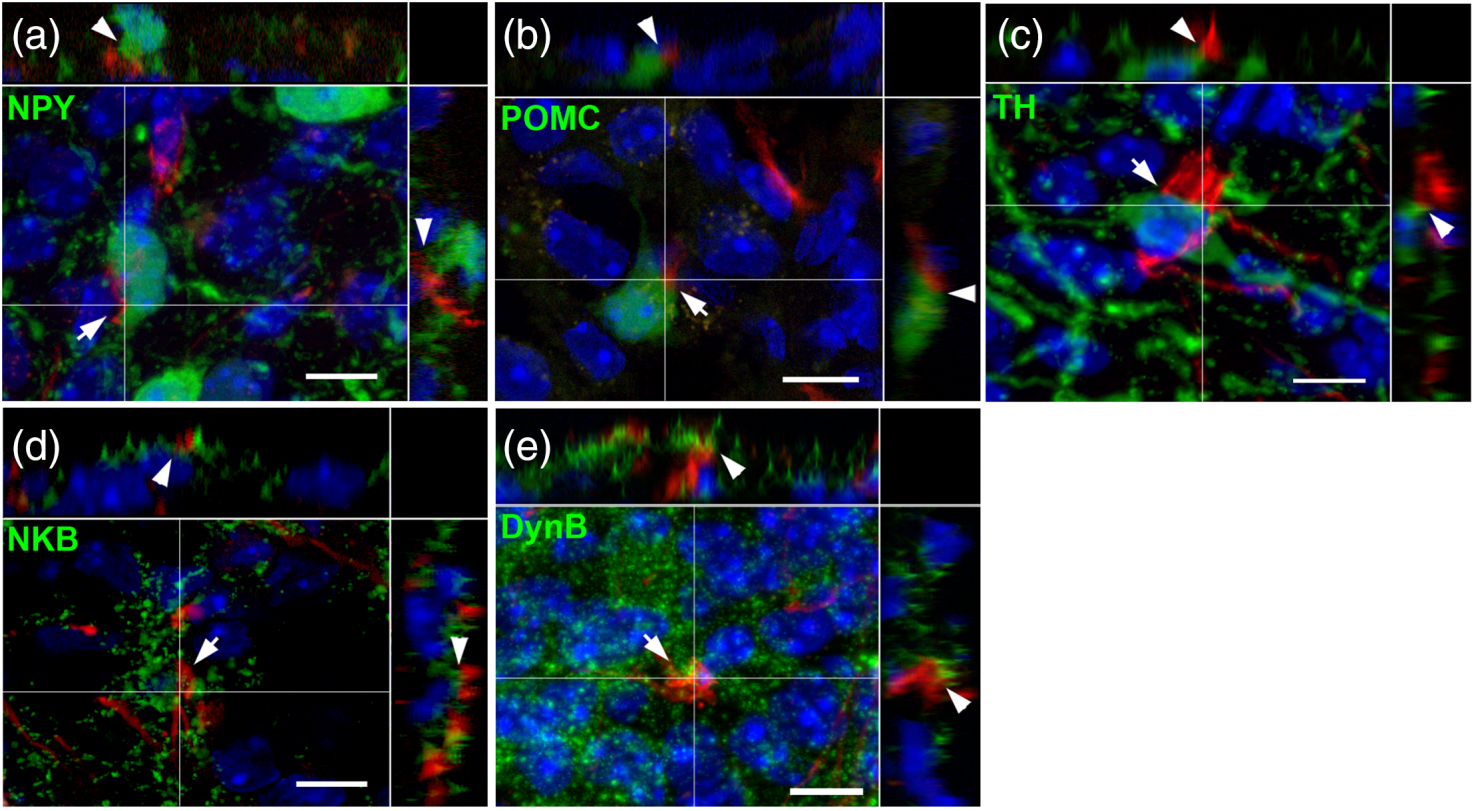
Tanycyte protrusions are in close proximity to ARH neurons. (a–e) High‐magnification z‐stack images (×63) showing the distribution of tdTomato (red, a–e), and NPY‐GFP fluorescence (green, a), POMC‐GFP fluorescence (green, b), tyrosine hydroxylase immunoreactivity (TH, green, c), neurokinine B immunoreactivity (NKB, green, d), and dynorphin B immunoreactivity (DynB, green, e), with DAPI counterstaining (blue, a–e) in coronal section in the arcuate nucleus, in Zone 2. Pictures (a–e) are the maximal intensity projections of z‐stack acquisition. Inset on the right in a–e panels shows the orthogonal view on the vertical line; inset on the top in a–e panels shows the orthogonal view on the horizontal line. Arrows and arrowheads indicate the site of contact in the coronal sections and in the orthogonal views, respectively. Low magnifications images are available upon request. Scale bars = 10 μm in a–e [Color figure can be viewed at wileyonlinelibrary.com]

As the entire ARH contains tanycyte processes, we next wanted to determine which arcuate neuronal populations are in contact with tanycyte protrusions. Both immunostaining and genetic mouse models reveal the presence of numerous contacts with NPY and POMC neurons (Figure 7a,b, respectively) (up to 24 and 10% association, respectively; Table 3), through tanycyte swellings as well as tanycyte boutons. A few contacts were also observed with TH‐positive neurons (Figure 7c), and KNDy neurons (Figure 7d,e).

### 3.6 Composition of tanycyte terminal boutons

**FIGURE 8:**
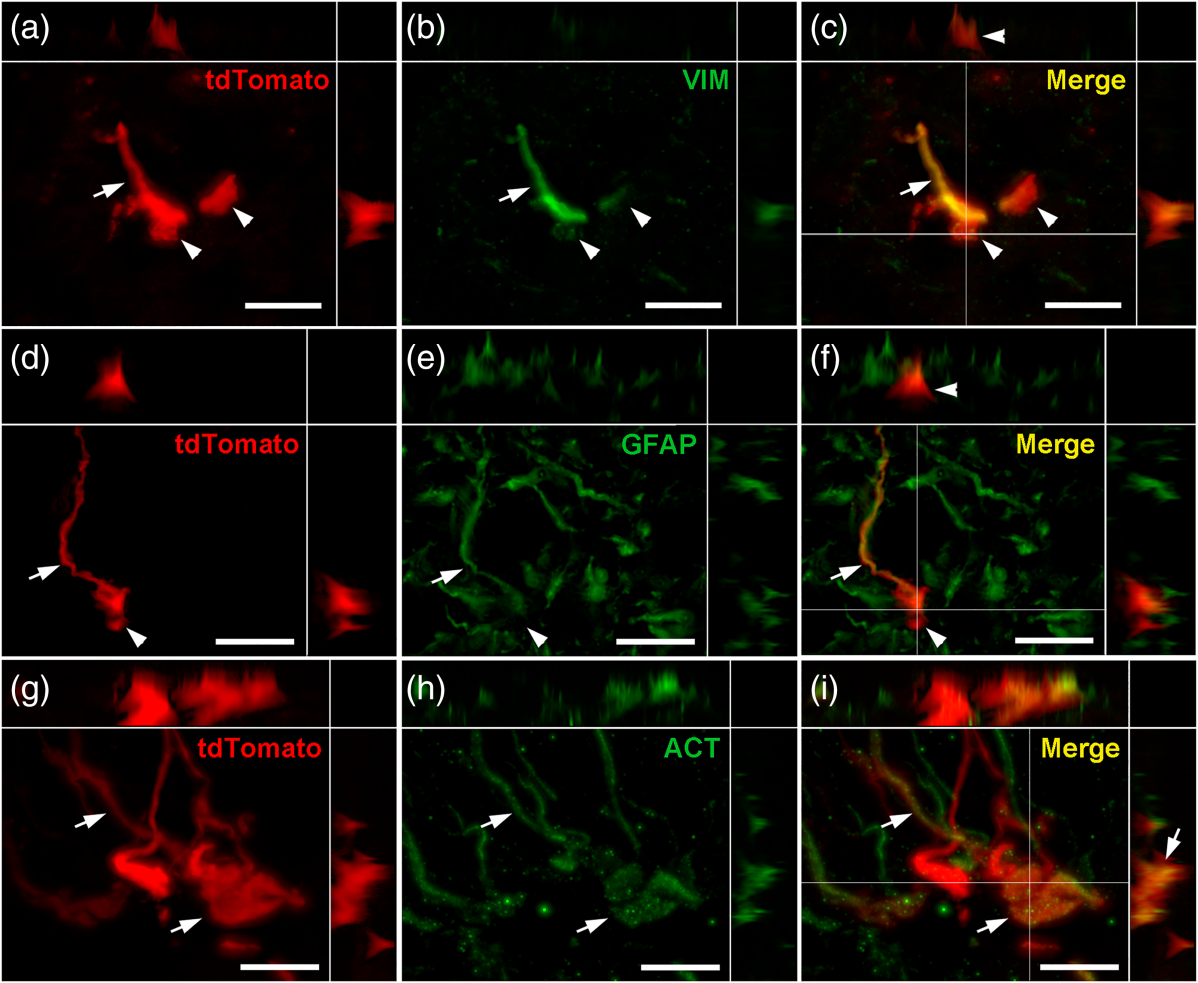
Cytoskeleton composition of tanycyte protrusions. (a–c) High‐magnification z‐stack images (×63) showing the distribution of tdTomato (red, a), vimentin immunoreactivity (VIM, green, b) and merge (yellow, c) in coronal section in the arcuate nucleus, in Zone 2. (d–f) High‐magnification z‐stack images (×63) showing the distribution of tdTomato (red, d), GFAP immunoreactivity (green, e) and merge (yellow, f) in coronal section in the arcuate nucleus, in Zone 2. (g–i) High‐magnification z‐stack images (×63) showing the distribution of tdTomato (red, g), actin immunoreactivity (ACT, green, h) and merge (yellow, i) in coronal section in the arcuate nucleus, in Zone 2. Pictures (a–i) are single planes of z‐stack acquisition. Inset on the top in a–i panels shows the orthogonal view on the horizontal line; inset on the right in a–i panels shows the orthogonal view on the vertical line. Arrows and arrowheads indicate colocalization observed or not, respectively, in tanycytes. Low magnifications images are available upon request. Scale bars = 10 μm in a–i [Color figure can be viewed at wileyonlinelibrary.com]

**FIGURE 9:**
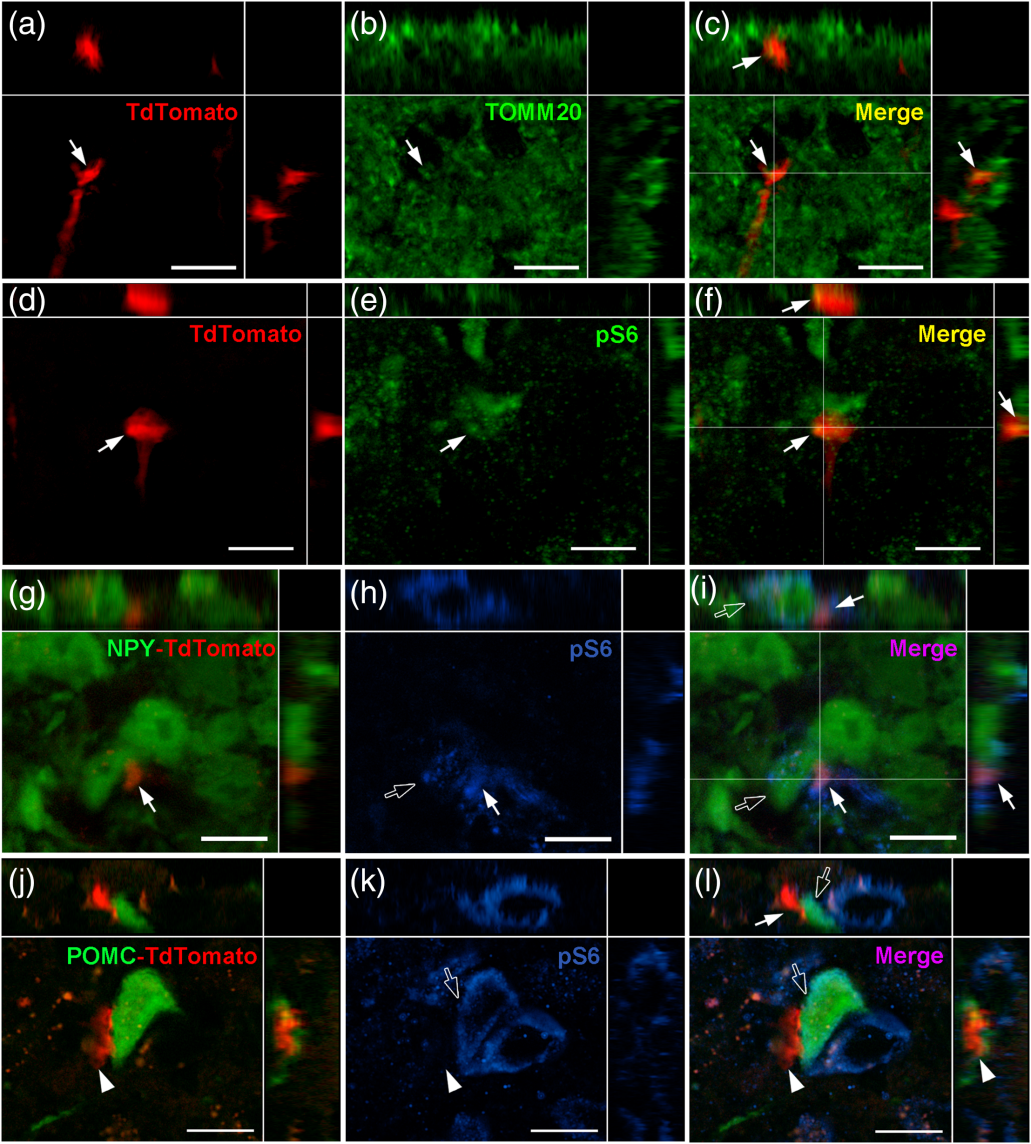
Organelle composition of tanycyte protrusions. (a–c) High‐magnification z‐stack images (×63) showing the distribution of tdTomato (red, a), TOMM20 immunoreactivity (green, b) and merge (yellow, c) in coronal section in the arcuate nucleus, in Zone 2. (d–f) High‐magnification z‐stack images (×63) showing the distribution of tdTomato (red, d), phospho‐S6 immunoreactivity (pS6, green, e) and merge (yellow, f) in coronal section in the arcuate nucleus, in Zone 2. (g–i**)** High‐magnification z‐stack images (×63) showing the distribution of tdTomato (red, g) and NPY‐GFP (green, g), pS6 immunoreactivity (blue, h) and merge (pink, i) in coronal section in the arcuate nucleus, in Zone 2. (j–l) High‐magnification z‐stack images (×63) showing the distribution of tdTomato (red, j) and POMC‐GFP (green, j), pS6 immunoreactivity (blue, k) and merge (pink, l) in coronal section in the arcuate nucleus, in Zone 2. Pictures (a–l) are single planes of z‐stack acquisition. Inset on the top in a–l panels shows the orthogonal view on the horizontal line; inset on the right in a–l panels shows the orthogonal view on the vertical line. Arrows and arrowheads indicate colocalization observed or not, respectively, in tanycytes. Empty arrows indicate colocalization observed in neurons. Low magnifications images are available upon request. Scale bars = 10 μm in a–l [Color figure can be viewed at wileyonlinelibrary.com]

**FIGURE 10:**
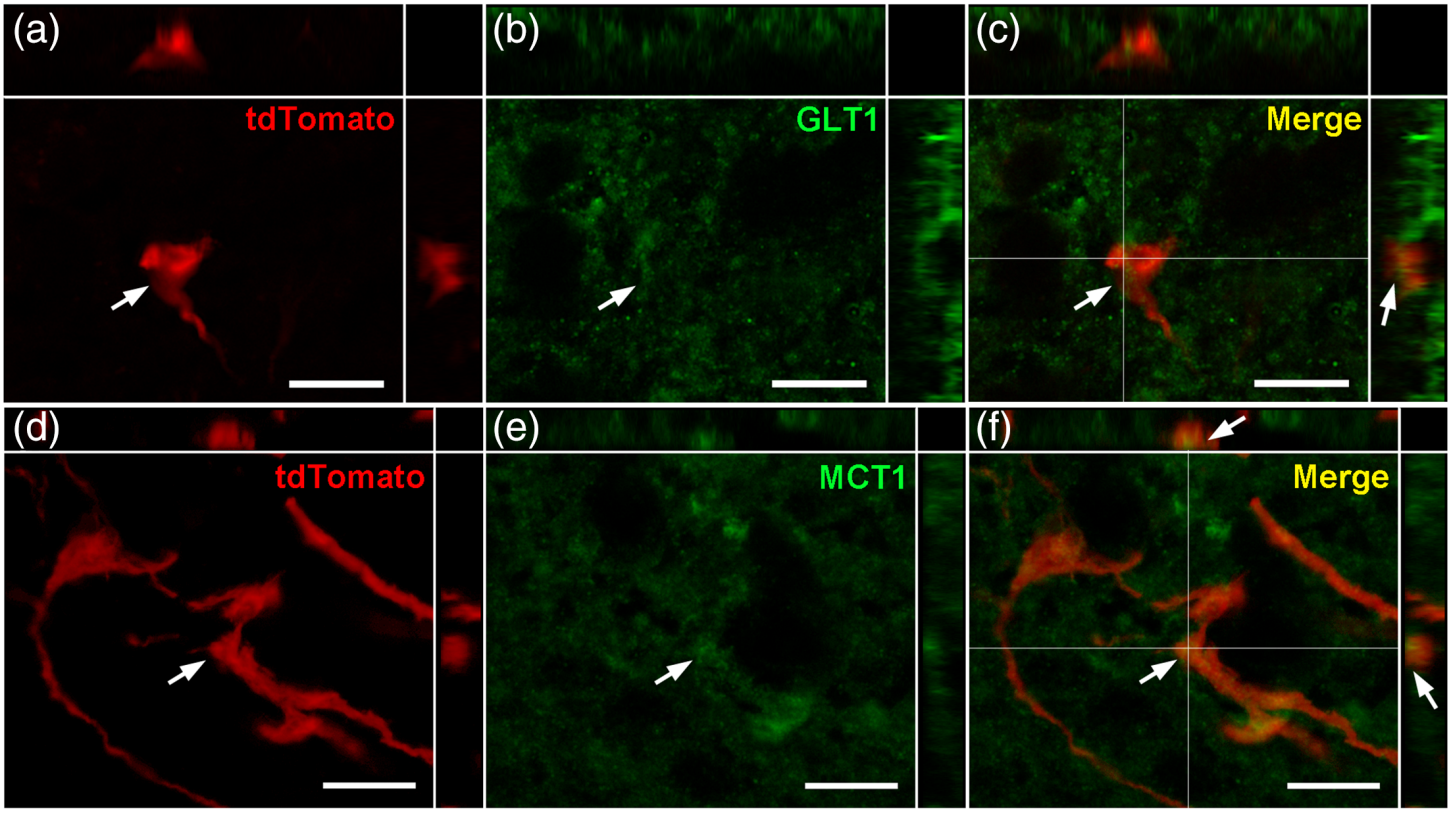
Transporter composition of tanycyte protrusions. (a–c) High‐magnification z‐stack images (×63) showing the distribution of tdTomato (red, a), GLT1 immunoreactivity (green, b) and merge (yellow, c) in coronal section in the arcuate nucleus, in Zone 2. (d–f) High‐magnification z‐stack images (×63) showing the distribution of tdTomato (red, d), MCT1 immunoreactivity (green, e) and merge (yellow, f) in coronal section in the arcuate nucleus, in Zone 2. Inset on the top in a–f panels shows the orthogonal view on the horizontal line; inset on the right in a–f panels shows the orthogonal view on the vertical line. Arrows indicate colocalization observed in tanycytes. Low magnifications images are available upon request. Scale bars = 10 μm in a–f [Color figure can be viewed at wileyonlinelibrary.com]

**FIGURE 11:**
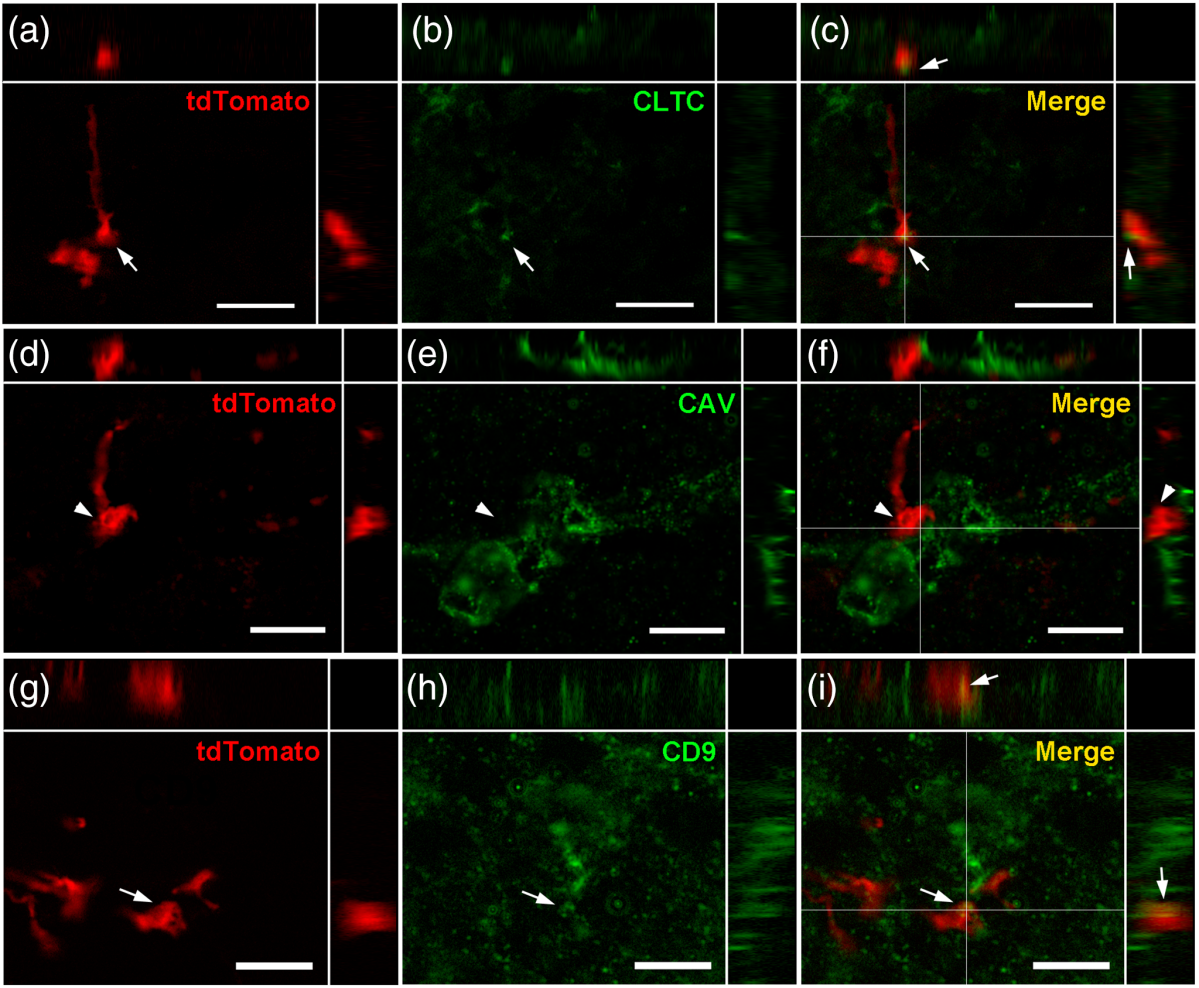
Vesicular system composition of tanycyte protrusions. (a–c) High‐magnification z‐stack images (×63) showing the distribution of tdTomato (red, a), clathrin immunoreactivity (CLTC, green, b) and merge (yellow, c) in coronal section in the arcuate nucleus, in Zone 2. (d–f) High‐magnification z‐stack images (×63) showing the distribution of tdTomato (red, d), caveolin immunoreactivity (CAV, green, e) and merge (yellow, f) in coronal section in the arcuate nucleus, in Zone 2. (g–i) High‐magnification z‐stack images (×63) showing the distribution of tdTomato (red, g), CD9 immunoreactivity (green, h) and merge (yellow, i) in coronal section in the arcuate nucleus, in Zone 2. Pictures (a‐i) are single planes of z‐stack acquisition. Inset on the top in a‐i panels shows the orthogonal view on the horizontal line; inset on the right in a–i panels shows the orthogonal view on the vertical line. Arrows and arrowheads indicate colocalization observed or not, respectively, in tanycytes. Low magnifications images are available upon request. Scale bars = 10 μm in a–i [Color figure can be viewed at wileyonlinelibrary.com]

To understand the putative function of these tanycyte protrusions, we next examined their composition using immunohistochemistry (Figures 8, 9, 10, 11).

Labelings for vimentin, GFAP, and actin were first performed to identify the cytoskeleton proteins present in tanycyte boutons (Figure 8). The intermediate filament protein vimentin is mainly located in the cell body and in the process, but is absent in the boutons (Figure 8a–c), explaining why labeling for vimentin, commonly used to visualize tanycytes, never allowed us to observe tanycyte protrusions before. The intermediate filament protein GFAP is expressed in some tanycyte subpopulations, mainly those facing the dmARH and VMH (Langlet, 2019). When present in tanycytes, GFAP is observed in the process, but not in boutons (Figure 8d–f). The microfilament actin is not detected in tanycyte cell bodies or processes, whereas it is in the distal process and in boutons of some vmARH tanycytes ending at the pial surface (Figure 8g–i).

Concerning organelles, mitochondria are detected in tanycyte boutons using TOMM20 immunostaining (Figure 9a–c). Translating ribosomes—observed using the phospho‐S6 marker—are present in tanycyte endfeet (Figure 9d–f), in particular in boutons contacting NPY (Figure 9g–i) and POMC neurons (Figure 9j–l). Interestingly, some of these contacted neurons also contain translating ribosomes (Figure 9g–l).

As α‐tanycytes are considered as modulators of neuronal activity (Coppola et al., 2007; Lanfray et al., 2013), we also evaluated the presence of neuromodulator transporters. Glial glutamate transporter GLT1 (Figure 10a–c) and lactate transporter MCT1 (Figure 10d–f) are located in some tanycyte boutons, suggesting a role for tanycyte protrusions in gliotransmission.

Finally, as tanycytes are involved in transport activity (Balland et al., 2014; Collden et al., 2015), we sought for vesicular system markers (i.e., caveolin, clathrin, CD9). Clathrin is located in some tanycyte boutons (Figure 11a–c), whereas caveolin is only present in blood–brain barrier (BBB) vessels (data not shown) but not in tanycyte endfeet (Figure 11d–f). As previously described (Horiguchi et al., 2019), exosomal marker CD9 is expressed by tanycytes: interestingly, CD9 is located in tanycyte endfeet (Figure 11g–i).

### 3.7 Ultrastructural characterization of tanycyte terminal boutons

**FIGURE 12:**
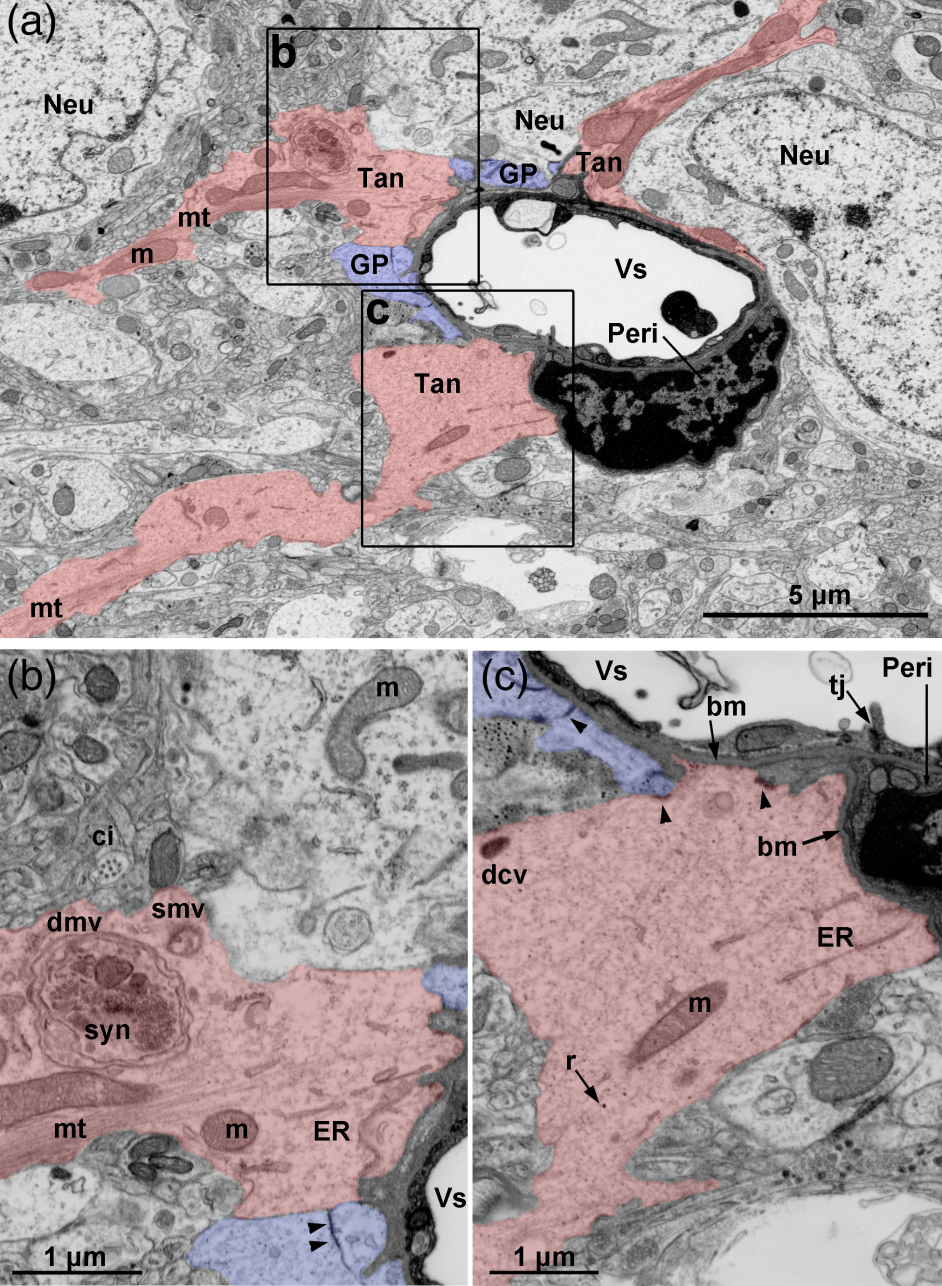
Fine structure of tanycyte endfeet. (a) Inverse contrast scanning electron microscopy micrograph showing the ultrastructure of the distal process and endfoot of three α‐tanycytes (tan, red) contacting a blood vessel (Vs) in the compact DMH in Zone 3 in adult male mice. Distal processes are filled with microtubules (mt) in their center, and display dilated segments containing elongated mitochondria (m). Around the blood vessel, tanycytes share the surface with glial processes (GP, violet) –probably belonging to astrocytes or tanycytes– and pericytes (Peri). Tanycytes also insulate neurons (Neu) from blood vessel. (b,c) Area of contact with the blood–brain barrier vessel and the pericyte. At the endfoot, mitochondria (m), ribosomes (r) and cisternae of the smooth endoplasmic reticulum and rough endoplasmic reticulum (ER) are present, but no microtubules. Double‐membrane vesicles (dmv) surrounding a synapse (syn), single‐membrane vesicles (smv), as well as dense‐core vesicles (dcv) are observed. Around the blood vessel (Vs), tanycytes are in contact with the basal lamina (bm), and establish interactions through junctional complexes (arrowheads) with pericytes and other glial processes. Tanycytes are also observed in contact with neuronal cell body close to primary cilia (ci). TJ, tight junctions. Scale bars: 5 μm in a, 1 μm in b,c. Pictures are represented in 3D in Video S2 [Color figure can be viewed at wileyonlinelibrary.com]

**FIGURE 13:**
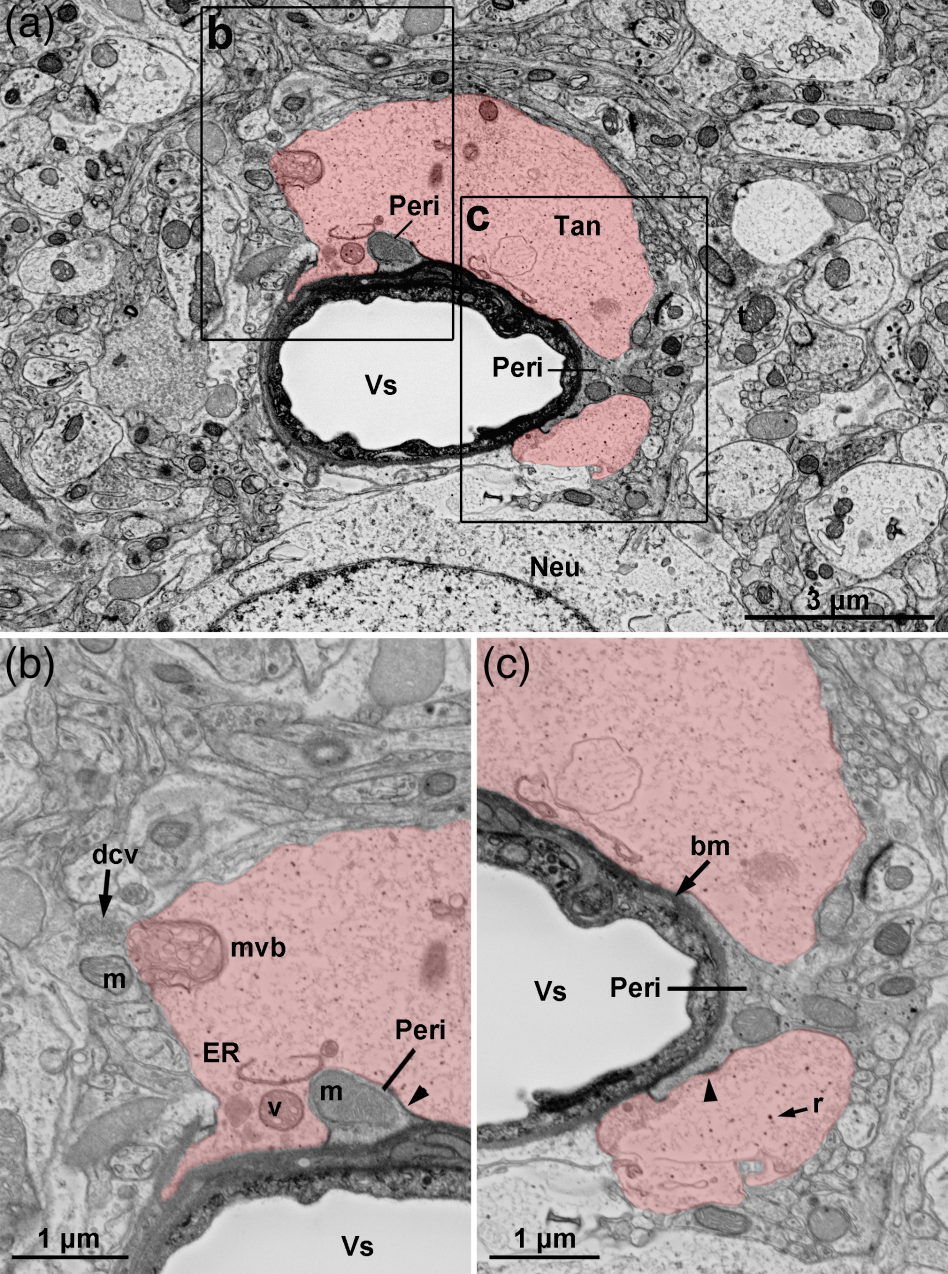
Vesicular system in tanycyte endfoot. (a) Inverse contrast scanning electron microscopy micrograph showing the ultrastructure of the distal process and endfoot of α‐tanycytes (tan, red) contacting a blood vessel (Vs) in the compact DMH in Zone 3 in adult male mice. (b,c) Area of contact with the blood–brain barrier vessel and the pericytes (Peri). At the endfoot, ribosomes (r), cisternae of endoplasmic reticulum (ER), dense vesicles (v) are present. Multivesicular bodies are also observed in tanycyte endfoot at the interface with brain parenchyma. Around the blood vessel (Vs), tanycytes are in contact with the basal lamina (bm), and establish interactions through junctional complexes (arrowheads) with other glial processes. Neu, neuron; m, mitochondria; dcv, dense core vesicles. Scale bars: 3 μm in a, 1 μm in b–c. Pictures are represented in 3D in Video S3 [Color figure can be viewed at wileyonlinelibrary.com]

**FIGURE 14:**
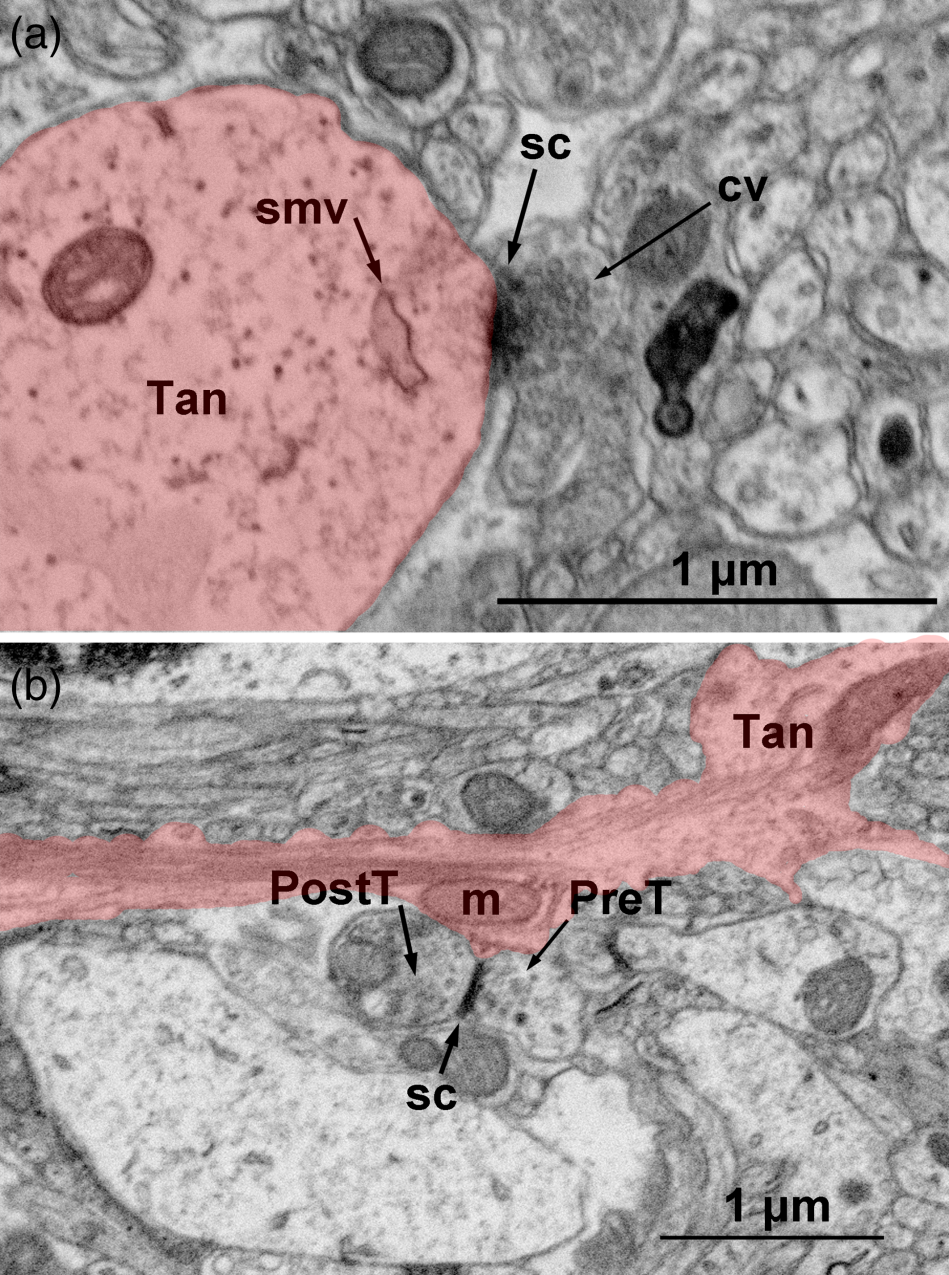
Ultrastructure of tanycyte/neuronal terminal associations. (a) Inverse contrast scanning electron microscopy micrograph showing a *synaptoid* contact on tanycyte endfoot (tan, red): This s*ynaptoid* contact is characterized by a presynaptic cytoplasm filled with small clear vesicles (cv), a synaptic cleft (sc) filled with an electron‐dense material, and a clear “postsynaptic” cytoplasm containing a single membrane vesicle (smv). Pictures are represented in 3D in Video S3. (b) Inverse contrast scanning electron microscopy micrograph showing a synapse surrounding by tanycyte endfoot (tan, red): This synapse is characterized by a vesicle‐rich presynaptic terminal (PreT), a synaptic cleft (sc), and a postsynaptic terminal (PostT), and is surrounded by a tanycyte process containing mitochondria. Pictures are represented in 3D in Video S3. Scale bars: 1 μm in a,b [Color figure can be viewed at wileyonlinelibrary.com]

To support our immunohistochemical observations, we finally examined tanycyte terminal boutons using electron microscopy (Figures 12, 13, 14, S2, and Videos S2,S3). Our regions of interest were defined before cutting by applying a semi‐correlative approach (Kolotuev, 2014): we combined the images of tdTomato fluorescent acquired prior sample preparation with the images of the embedded samples. After sectioning, we found tanycyte endfeet throughout the brain parenchyma using morphological landmarks (i.e., ventricle and blood vessels), as well as ultrastructural characteristics of neural cells previously reported in the literature (Luse, 1956; Rodríguez et al., 2019) (Figure S2). Once tanycyte endfeet were recognized, the acquisition was done for the complete series of sections spanning a significant volume to finally build 3D reconstructions of tanycyte process and endfoot (Burel et al., 2018) (Videos S2 and S3).

As reported in our morphometric analysis, tanycyte processes are ~1 μm thick, display some dilated portions and form ~5 μm wide bouton‐like endfeet (Figure 12a, Videos S2 and S3). These tanycyte boutons contact the basal lamina surrounding BBB microvessels, where they share the surface with other tanycyte endfeet (Figure 12), glial endfeet as well as pericytes (Figures 12, 13). Interestingly, electron densities corresponding to junctional complexes are located at the site of contacts between tanycytes, glial cells, and pericytes (arrows in Figures 12 and 13).

The fine structure of tanycyte is easily recognizable within brain parenchyma by their clear cytoplasm. Microtubules are present along the process but are missing at the endfeet (Figure 12), comparable with our observation for the intermediate filament protein vimentin and GFAP (Figure 8a–f). In contrast, the endfeet mainly appear to be filled with a filamentous material (Figures 12 and 13). Among the cytoplasm material, elongated mitochondria run along the process and are present at the endfeet (Figure 12). The endfeet also contain a variable number of ribosomes, and cisternae of endoplasmic reticulum (Figures 12 and 13). Interestingly, endoplasmic reticulum is mainly smooth, and closely contact the plasma membrane (Video S2). Finally, the main feature of tanycyte endfeet relies on the diversity of their vesicular system (Figures 12 and 13). Indeed, phagosome, double‐membrane vesicles, single membrane vesicles, multivesicular bodies, as well as dense‐core vesicles are present in tanycyte boutons (Figures 12 and 13). In particular, we report here multivesicular bodies at the interface with brain parenchyma, in particular close to mitochondria (Figure 13b, Video S3).

Our electron microscopy 3D reconstructions lastly reveal peculiar associations with neurons. First, tanycyte endfeet directly contact neuronal cell body, close to their primary cilia, and separate them from blood vessels (Figure 12b, Video S2), as astrocyte endfeet do at the BBB. Moreover, as previously reported for tanycytes (Rodríguez et al., 2019) and astrocytes, neurons and tanycytes establish special connections through *synaptoid contacts* (Figure 14a, Video S3). They are characterized by a presynaptic cytoplasm filled with 35 nm small clear vesicles, a synaptic cleft filled with an electron‐dense material, and a “postsynaptic” cytoplasm being either empty or with some organelles (Figure 14a). Moreover, tanycytes are also in close proximity with synapses. Indeed, numerous synapses characterized by a vesicle‐rich presynaptic terminal, a synaptic cleft, and a postsynaptic density, are observed throughout the sections. Some of them are surrounded by tanycyte processes and endfeet (Figure 14b). Interestingly, mitochondria may be observed at these sites in tanycyte cytoplasm (Figure 14b, Video S2). Finally, tanycyte endfeet also encapsulate synapses (Figure 12b, Video S2), confirming our observation using fluorescent microscopy (Figure 6a–f).

## 4 DISCUSSION

In the present study, we used genetic approaches to express the fluorescent tdTomato protein in tanycytes in order to systematically examine their morphology. In contrast to vimentin immunostaining, this approach allowed us to reveal the presence of peculiar protrusions along tanycytes processes, contacting diverse neural cells throughout the hypothalamic parenchyma.

In the literature, similar protrusions were previously described along tanycyte processes in different species using Golgi‐cox impregnation (Bleier, 1971; Card & Rafols, 1978; Fasolo & Franzoni, 1974; Joy & Sathyanesan, 1981; Millhouse, 1971). From amphibians to mammals, tanycytes are similar enough to lead to a classic generalization about their structure: they morphologically resemble the embryonic radial glial cells composed of a somatic region, a long process, and an endfoot. Unusual elements exist along their process: they are described as slim or large, smooth or beaded, tortuous or straight, sparsely or densely spinous as well as branched or unbranched, and with different types of endfeet. Besides these similarities, tanycytes display specific characteristics for each species. First, tanycytes are more abundant in the brains of nonmammalian vertebrates (i.e., adult fish, amphibians, and reptiles) (Fasolo & Franzoni, 1974), but restricted to few localized brain areas in birds and mammals (Bleier, 1971). In humans, they are mainly found in the anterior and middle part of the infundibular and median eminence region (Koopman, Taziaux, & Bakker, 2017). Secondly, the density of protrusions along tanycyte processes also varies according to the species. Tanycytes are more spinous and tortuous in amphibians compared with mammals (Bleier, 1971; Fasolo & Franzoni, 1974). Here, we confirm the presence of such protrusions along tanycyte processes in adult male mice. Like in other mammals, these protrusions are less abundant than in amphibians or reptiles: tanycyte processes are quite straight and their protrusions are mainly located at the proximal (i.e., spines) and the distal portions (i.e., swellings, boutons). Interestingly, differences exist between mice and rats (Bleier, 1971; Joy & Sathyanesan, 1981; Millhouse, 1971), although these two species are closely related to one another. In particular, spines in the neck region are more numerous in the rat and present up to the median eminence, whereas they are restricted to the DMH and VMH in mice.

The present study also shows that, besides interspecies differences, tanycyte morphology differs within the same animal, revealing an inadequacy in the current tanycyte classification (i.e., *β1*, *β2*, *α1*, and *α2*). Along the ventrodorsal axis, at least four morphologically distinct tanycyte populations would line the lateral wall of the third ventricle, facing respectively in the vmARH, the dmARH, the VMH and the DMH. Indeed, tanycytes lining the DMH and the VMH—currently called α1‐tanycytes—present differences regarding the morphology of their cell body as well as their protrusions (i.e., boutons and swellings). Moreover, morphological differences between tanycytes facing the dmARH and vmARH are striking, especially regarding their protrusions (i.e., spines) and their endfeet (i.e., endfeet within the parenchyma or at the pial surface). However, a clear delimitation between these different subgroups remains uneasy to make: indeed, some protrusions, such as spines present at the neck portion along the VMH and DMH, progressively disappear in the ARH, describing a ventrodorsal “gradient” rather than a feature of clear‐cut tanycyte subgroups. Additionally, our study describes morphological differences from tanycyte to tanycyte along the anteroposterior axis, especially concerning their diameter and the direction of their processes. Therefore, as discussed previously (Langlet, 2019), improving tanycyte classification taking into account both the anteroposterior and ventrodorsal axis is crucial and constitutes our next challenge to further understand tanycyte biology.

In contrast to Golgi's method that stains many different neural cell types at random (Bleier, 1971; Card & Rafols, 1978; Fasolo & Franzoni, 1974; Joy & Sathyanesan, 1981; Millhouse, 1971), our approach inducing tdTomato expression in the ependymal layer allows us to visualize with certainty the interactions between tanycytes and other neural cells present in the hypothalamic parenchyma. In this study, we first show that, while tanycytes contact each other mainly through their cell bodies, spine‐to‐spine and endfeet‐to‐endfeet contacts were also detected between distinct tanycytes at the proximal and distal region of their processes, respectively. The formation of these close contacts confirm the importance of tanycyte‐to‐tanycyte communications, presumably for the synchronization of their functions. These communications could occur through gap junction protein Connexin‐43 given that its selective depletion in astrocytes and tanycytes disrupts tanycyte‐coupled network (Recabal et al., 2018). Secondly, this study settles that tanycytes are in contact with multiple other neural cells. Their main heterotypic partners confirmed in our study are blood vessels. This tanycyte/blood vessel relationship was largely described in the literature (Rodríguez et al., 2019): linking the blood and the cerebrospinal fluid, tanycytes are therefore described as gateways to the brain (Langlet, 2014). Here, we also observe that many tanycyte endfeet share the space around the blood vessels with pericytes and astrocytes: multiple way exchanges between these different cell types, in particular for the formation and maintenance of the hypothalamic blood–brain barrier, remain to be explored. Contacts with other glial cells such as oligodendrocytes are also present, confirming previous reports (Recabal et al., 2018). Additionally, we describe here associations with different neuronal populations, suggesting that tanycytes integrate into multiple neuronal networks, resulting in the regulation of diverse physiological functions. In particular, tanycytes contact NPY and POMC neuronal populations as well as KNDy neurons: such associations likely play a role in the regulation of energy balance and reproduction, respectively. So far, tanycyte/neuron interactions were a matter of controversy in the literature. Indeed, with Golgi preparations, some authors found the presence of such an ependymo–neuron contact (Bleier, 1971), whereas others did not (Card & Rafols, 1978; Millhouse, 1971; Rodríguez et al., 2019). Close anatomical contacts with NPY neurons were already reported (Coppola et al., 2007), but those with other neuronal populations were never described before. Besides neuronal cell bodies, our study also reveals tanycyte associations with GABAergic terminals and glutamatergic terminals. The first type of associations consists of *synaptoid* contacts on tanycyte processes as previously reported in rats for β and α tanycytes (Rodríguez et al., 2019; Rodríguez et al., 2005). The functional significance of these *synaptoid* contacts remains to be elucidated, but suggests that tanycyte/neuron communications are likely bi‐directional. By this way, neurons could control tanycyte functions, as it was previously hypothesized for the regulation of volume transmission (Alpár, Benevento, Romanov, Hökfelt, & Harkany, 2019). Here, we additionally provide the description of tanycyte protrusions surrounding synapses suggesting a role for tanycytes in the modulation of synaptic transmission. This hypothesis is strengthened by the presence of GLT1 glutamate transporter in tanycyte boutons necessary to control extracellular glutamate in hypothalamic nuclei, and to ensure brain homeostasis and synaptic transmission. Further experiments are consequently needed to analyze tanycyte proximity with synapses as well as their plasticity, and to evaluate if tanycytes, mimicking astrocyte function, could play a role in functional tripartite synapses. Finally, we also show that tanycyte endfeet encapsulate synapses, suggesting a role for tanycytes in synapse elimination. While phagocytosis is primarily attributed to microglia, it also was described in other glial cells such as astrocytes or oligodendrocytes in order to maintain homeostasis in the brain (Lee & Chung, 2019). By this way, tanycytes could contribute to synaptic plasticity.

Although we focused our study on the ARH, these different tanycyte partners were also observed in the VMH and DMH, suggesting that tanycytes are involved in different neural network regulating diverse physiological functions. Moreover, tanycytes‐like cells being present in other brain areas (Langlet, Mullier, et al., 2013), similar contacts could also be observed in these regions: some differences and/or specificity between brain areas cannot be nevertheless excluded. Finally, it is worth to note that these different partners could impact tanycyte morphology per se. Indeed, cells sense the presence of interaction partners and, specifically respond by changing the expression of many target genes resulting in a specific cell morphology adapted to their functions and communications. Therefore, the existence of different neuronal populations with different anteroposterior and ventrodorsal distributions within the same nucleus may influence tanycyte morphology and functions on both axes. Based on that, we can consequently predict that a higher number of different tanycyte subgroups exist.

The numerous protrusions present along tanycyte processes and contacting neurons and/or blood vessels strongly suggest a functional relationship between these cell types. The analysis of their composition allows us to speculate about this relationship. The presence of different organelles, including ribosomes and mitochondria, in tanycyte protrusions first indicates that they are active subcellular domains along tanycyte processes. In particular, we observed phosphorylated ribosomal protein S6 in tanycyte boutons suggesting local translation. Interestingly, ribosomes are also translating in neurons facing these tanycyte boutons suggesting a parallel activity. Additionally, numerous endoplasmic reticulum‐plasma membrane (ER‐PM) contacts were observed in tanycyte protrusions. These structures could play a critical role in the integration of central or/and peripheral information. Indeed, ER‐PM contacts serve as important sites for cellular signaling pathways including lipid and calcium signaling, and metabolic regulation. Neural and peripheral signals received by tanycytes at their protrusions could by this way regulate numerous downstream signaling effectors and modulate tanycyte function both locally and globally. In keeping with the multiple evidences suggesting that tanycytes transport materials between the CSF and the blood and vice versa, our study also reports the presence of diverse vesicles including multivesicular bodies, phagosomes, double membrane vesicles, suggesting the presence of both normal and unconventional secretory pathways such as exosomes and secretory autophagy. However, further experiments are needed to understand the functional role of such different transport systems. Finally, this study also reports the presence of MCT1 lactate transporter in tanycyte boutons. While previous studies reported their expression in tanycytes (Berger & Hediger, 2001; Cortés‐Campos et al., 2011), ours shows their localization at tanycyte/neural cell interaction sites. The current hypothesis is that tanycytes would release lactate via MCT1 in order to influence the activity of neurons regulating food intake (Cortés‐Campos et al., 2011; Elizondo‐Vega et al., 2016; Elizondo‐Vega, Recabal, & Oyarce, 2019). Interestingly, arcuate neurons also express lactate transporters such as MCT1 (Carneiro et al., 2016) and MCT2 (Cortés‐Campos et al., 2011), suggesting that their activity could be regulated via lactate coming from tanycytes as part of the glucose‐sensing mechanism.

## 5 CONCLUSION

Based on this neuroanatomical study, we propose that tanycytes serve as a communication system between the cerebrospinal fluid, brain capillaries and neural cells within the hypothalamus. The different protrusions found along tanycyte processes would allow them to integrate information coming from the brain through the CSF and the periphery through blood vessel, and to redistribute it to neural cells throughout the hypothalamus.

## CONFLICT OF INTEREST

The authors have no conflict of interest to declare.

## Supporting information


**Supplemental Figure 1** **Subdivisions of the mediobasal hypothalamus on the anteroposterior axis.** Coordinates on the anteroposterior axis (a) and schematics of the four zones (b) used for morphometric analysis. Zone 1 corresponds to the anterior part of the ARH and the ME where tanycyte processes are mainly found in the ARH and few of them in the VMH. Zone 2 corresponds to the medial part of ME where the bottom of the ventricle is larger and tanycyte processes are found in both the ARH and VMH. Zone 3 corresponds to the medio‐posterior part of the ME, where the VMH is lateral and tanycyte processes are now observed in the DMH. Zone 4 corresponds to the posterior part of the ME and the presence of the infundibular stalk, where tanycyte processes are sent in the ARH and DMH. In b, red tanycytes represent tanycytes contacting the fenestrated blood vessels of the median eminence; yellow tanycytes represent tanycytes contacting the pial surface of the brain; and green tanycytes represent tanycytes contacting neural cells in the brain parenchyma. 3 V, third ventricle; ARH, arcuate nucleus; DMH, dorsomedial nucleus; ME, median eminence; VMH, ventromedial nucleus.

**Supplemental figure 2** **Methodology to improve our chances to find tanycyte endfeet on electron microscopy. a‐c,** Semi‐correlative approach consisting of superimposed pictures of Tdtomato fluorescence from vibratome slices (a) and pictures of the embedded samples (b), while keeping the same orientation. A region of interest containing tanycyte endfeet was then selected (Cutting area): the tissue around these regions was tightly trimmed and the sections that will potentially span the region were collected on the large surface wafers. **d‐f,** Tanycyte endfeet of interest were then localized within the cutting area using arbitrary landmarks (i.e. ependyma and blood vessels [Vs]) by measuring their depth within the slice (d) and their distance from the ependyma (e) and the blood vessels (f). **g‐h,** Orthogonal view of fluorescent (g) and electron microscopy pictures (h) finally allow us to localize tanycyte endfeet on electron microscopy pictures. 3 V, third ventricle; ARH, arcuate nucleus; DMH, dorsomedial nucleus. Scale bar in G = 100 μm in g.

**Supplemental Video 1** Video presenting tdTomato fluorescence in tanycytes, and showing spine‐to‐spine contacts between the proximal portion of two adjacent α‐tanycytes in the VMH in adult male mice.

**Supplemental Video 2** Video presenting inverse contrast scanning electron microscopy micrographs in the serial sections, and showing the distal process and endfoot of three α‐tanycytes in the compact DMH in Zone 3 in adult male mice.

**Supplemental Video 3** Video presenting inverse contrast scanning electron microscopy micrographs in the serial sections, and showing the distal process and endfoot of two α‐tanycytes in the compact DMH in Zone 3 in adult male mice.

## Data Availability

The data that support the findings of this study are available from the corresponding author upon reasonable request.
