# Supplementary figures and images for: Peculiar protrusions along tanycyte processes face diverse neural and nonneural cell types in the hypothalamic parenchyma
Source: J Comp Neurol. 2020 Jun 24;529(3):553–75. doi: 10.1002/cne.24965 (PMC7818493; doi:10.1002/cne.24965)

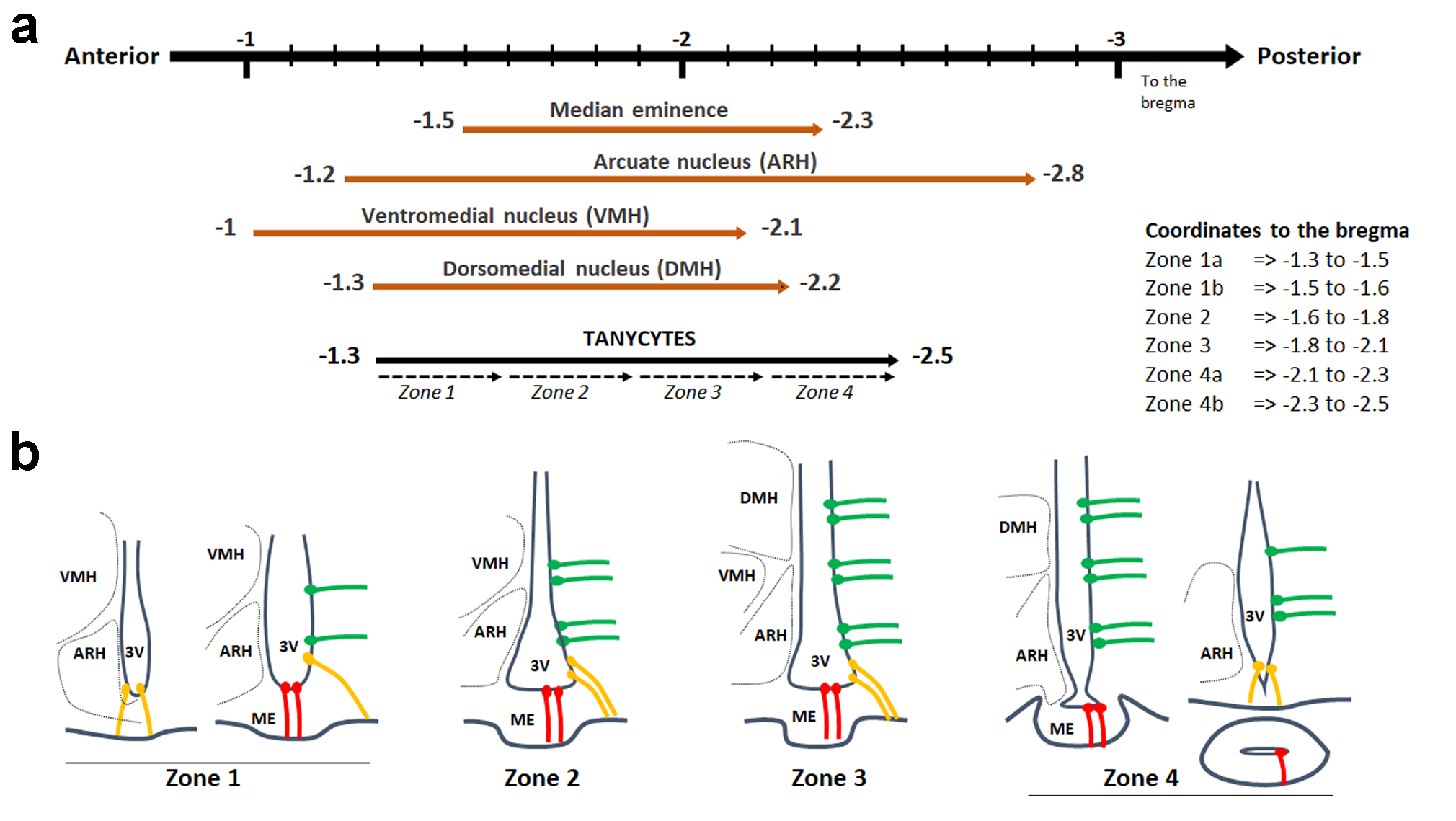

Supplement: Supplementary file 1 — Supplemental Figure 1 Subdivisions of the mediobasal hypothalamus on the anteroposterior axis. Coordinates on the anteroposterior axis (a) and schematics of the four zones (b) used for morphometric analysis. Zone 1 corresponds to the anterior part of the ARH and the ME where tanycyte processes are mainly found in the ARH and few of them in the VMH. Zone 2 corresponds to the medial part of ME where the bottom of the ventricle is larger and tanycyte processes are found in both the ARH and VMH. Zone 3 corresponds to the medio‐posterior part of the ME, where the VMH is lateral and tanycyte processes are now observed in the DMH. Zone 4 corresponds to the posterior part of the ME and the presence of the infundibular stalk, where tanycyte processes are sent in the ARH and DMH. In b, red tanycytes represent tanycytes contacting the fenestrated blood vessels of the median eminence; yellow tanycytes represent tanycytes contacting the pial surface of the brain; and green tanycytes represent tanycytes contacting neural cells in the brain parenchyma. 3 V, third ventricle; ARH, arcuate nucleus; DMH, dorsomedial nucleus; ME, median eminence; VMH, ventromedial nucleus. [file CNE-529-553-s001.tif]

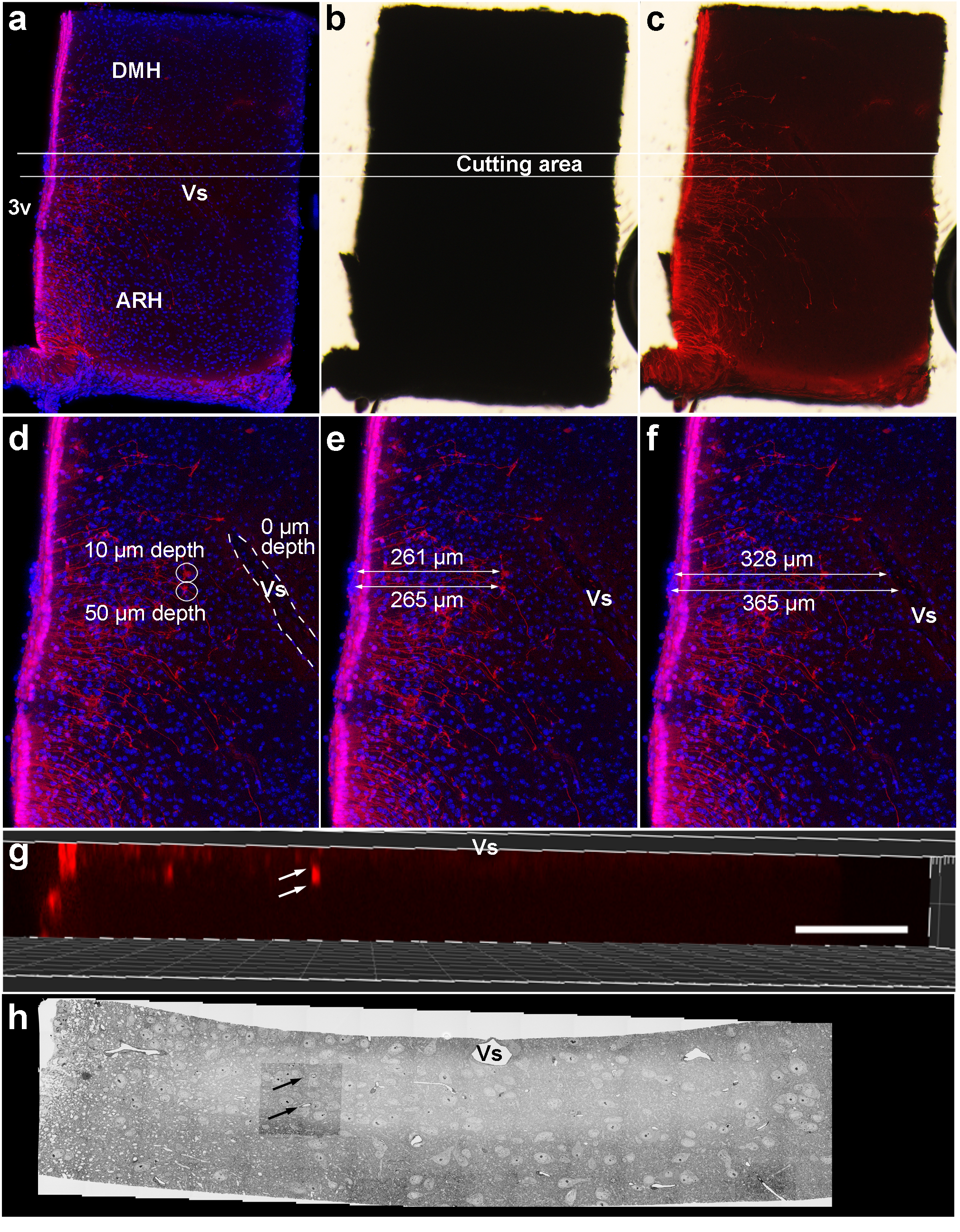

Supplement: Supplementary file 2 — Supplemental figure 2 Methodology to improve our chances to find tanycyte endfeet on electron microscopy. a‐c, Semi‐correlative approach consisting of superimposed pictures of Tdtomato fluorescence from vibratome slices (a) and pictures of the embedded samples (b), while keeping the same orientation. A region of interest containing tanycyte endfeet was then selected (Cutting area): the tissue around these regions was tightly trimmed and the sections that will potentially span the region were collected on the large surface wafers. d‐f, Tanycyte endfeet of interest were then localized within the cutting area using arbitrary landmarks (i.e. ependyma and blood vessels [Vs]) by measuring their depth within the slice (d) and their distance from the ependyma (e) and the blood vessels (f). g‐h, Orthogonal view of fluorescent (g) and electron microscopy pictures (h) finally allow us to localize tanycyte endfeet on electron microscopy pictures. 3 V, third ventricle; ARH, arcuate nucleus; DMH, dorsomedial nucleus. Scale bar in G = 100 μm in g. [file CNE-529-553-s002.tif]
